# Genetic and Ultrastructural Analysis Reveals the Key Players and Initial Steps of Bacterial Magnetosome Membrane Biogenesis

**DOI:** 10.1371/journal.pgen.1006101

**Published:** 2016-06-10

**Authors:** Oliver Raschdorf, Yvonne Forstner, Isabel Kolinko, René Uebe, Jürgen M. Plitzko, Dirk Schüler

**Affiliations:** 1 Department Biology I-Microbiology, Ludwig Maximilian University Munich, Planegg-Martinsried, Germany; 2 Department of Molecular Structural Biology, Max Planck Institute of Biochemistry, Planegg-Martinsried, Germany; 3 Department of Microbiology, University of Bayreuth, Bayreuth, Germany; Max Planck Institute for Terrestrial Microbiology, GERMANY

## Abstract

Magnetosomes of magnetotactic bacteria contain well-ordered nanocrystals for magnetic navigation and have recently emerged as the most sophisticated model system to study the formation of membrane bounded organelles in prokaryotes. Magnetosome biosynthesis is thought to begin with the formation of a dedicated compartment, the magnetosome membrane (MM), in which the biosynthesis of a magnetic mineral is strictly controlled. While the biomineralization of magnetosomes and their subsequent assembly into linear chains recently have become increasingly well studied, the molecular mechanisms and early stages involved in MM formation remained poorly understood. In the Alphaproteobacterium *Magnetospirillum gryphiswaldense*, approximately 30 genes were found to control magnetosome biosynthesis. By cryo-electron tomography of several key mutant strains we identified the gene complement controlling MM formation in this model organism. Whereas the putative magnetosomal iron transporter MamB was most crucial for the process and caused the most severe MM phenotype upon elimination, MamM, MamQ and MamL were also required for the formation of wild-type-like MMs. A subset of seven genes (*mamLQBIEMO*) combined within a synthetic operon was sufficient to restore the formation of intracellular membranes in the absence of other genes from the key *mamAB* operon. Tracking of *de novo* magnetosome membrane formation by genetic induction revealed that magnetosomes originate from unspecific cytoplasmic membrane locations before alignment into coherent chains. Our results indicate that no single factor alone is essential for MM formation, which instead is orchestrated by the cumulative action of several magnetosome proteins.

## Author Summary

One of the most intriguing examples for membrane-bounded prokaryotic organelles are magnetosomes which consist of well-ordered chains of perfectly shaped magnetic nanocrystals that in many aquatic bacteria serve as geomagnetic field sensors to direct their swimming towards microoxic zones at the bottom of natural waters. In the model bacterium *Magnetospirillum gryphiswaldense* and related magnetotactic microorganisms, magnetosomes are formed by a complex pathway that is orchestrated by more than 30 genes. However, the initial and most crucial step of magnetosome biosynthesis, formation and differentiation of a dedicated intracellular membrane compartment for controlled biomineralization of magnetite crystals, remained only poorly understood. By ultrastructural analysis of several mutants and genetic induction of *de novo* magnetosome synthesis, we identified the key determinants and early steps of magnetosome membrane biogenesis. Our results suggest that formation of intracellular membranes in bacteria is mediated by a cumulative action of several factors, but apparently is differently controlled than intracellular membrane remodeling in eukaryotic cells.

## Introduction

Although many prokaryotes are currently known to form intracytoplasmic membranes (ICM), the underlying mechanisms of ICM formation are still widely unexplored [1,2]. One of the most sophisticated model systems to study the biogenesis of prokaryotic ICM and organelles are bacterial magnetosomes, which are nanometer-sized particles of a magnetic mineral bounded by a distinct membrane [3,4]. In the freshwater Alphaproteobacterium *Magnetospirillum gryphiswaldense* (MSR-1), biosynthesis of magnetosomes was dissected into distinct steps. First, by invagination from the cytoplasmic membrane (CM) the magnetosome membrane (MM) forms a confined compartment. Next, supersaturating amounts of iron are transported into the MM which provides a controlled environment for the nucleation and maturation of well-ordered cuboctahedral crystals of magnetite. Eventually, individual magnetosome particles become concatenated and aligned into a linear chain along a dedicated cytoskeletal structure to most efficiently serve as sensor for the Earth’s weak magnetic field [5–8]. While the biomineralization of magnetite crystals is increasingly well understood, much less is known about the genetic determinants and mechanisms which control the formation of the MM. In MSR-1 the MM was found to contain a set of specific proteins [9,10] which are assumed to control magnetosome biosynthesis and are encoded by several gene clusters of a compact genomic magnetosome island (MAI) [11–13] (S1 Fig). Previous studies of MSR-1 suggested that only 6 (*mamB*, *mamM*, *mamE*, *mamO*, *mamQ*, and *mamL*) of the ~30 known MAI genes are individually essential for the biomineralization of at least rudimentary iron-oxide particles as revealed by conventional TEM. MamQ belongs to the widespread LemA protein family [10], but magnetobacterial MamQ is the only member that could be linked to a known cellular process [14]. MamL is a small, MTB-specific hypothetical membrane protein without any known functional domains [15]. The paralogous MamB and MamM proteins belong to the cation diffusion facilitator family and are assumed to transport ferrous iron into the magnetosome lumen. Both proteins form homo- and heterodimers and MamB becomes destabilized in the absence of MamM [8]. It was further suggested that the C-terminal tetratricopeptide repeat domain of MamB interacts with the PDZ domain of MamE [8]. MamE and MamO are putative membrane-integral serine proteases or degenerated members of the HtrA/DegP family and important for magnetite maturation *in vivo* [16,17]. It was suggested that MamE functions in magnetosome protein sorting, and by its protease activity provides a “checkpoint” control for magnetite maturation [16].

While deletion of *mamB* in MSR-1 reportedly also eliminated the formation of MMs, Δ*mamM*, Δ*mamE* and Δ*mamO* strains continued to form empty MM vesicles devoid of electron dense crystals [8,18]. In the related *Magnetospirillum magneticum* (AMB-1) in addition single deletions of *mamL*, *mamQ* and *mamI* (encoding a small MTB-specific protein of unknown function) were reported to completely eliminate MM formation, while deletion of *mamN* (encoding a putative proton exporter) caused the formation of empty MMs [19]. However, the effects on MM formation have not been studied in Δ*mamQ*, Δ*mamN*, Δ*mamI* and Δ*mamL* mutant strains of MSR-1, and thus the full complement of genes and their specific functions controlling MM formation in MTB has remained unknown. In addition, it is not clear whether MM formation proceeds in a stepwise manner, and if so, whether intermediate MM states of invagination exist, and how magnetosome proteins become organized prior and during MM formation. It is further still disputed whether the invaginated MMs remain permanently connected with the CM, eventually become pinched off to form free magnetosome vesicles, or if different MTB use different mechanisms [20].

In this study, we thoroughly characterized magnetosome membranes in MSR-1 wild type and several mutant strains. Cryo-electron tomography (CET) revealed previously unknown details of MM structure, including the presence of novel atypical membrane vesicles, putatively representing defective or immature MM states. We systematically assessed the gene complement controlling MM formation and discovered that MamB is the most crucial protein for MM formation. While the combined expression of all identified genes affecting MM formation (*mamLMQB)* failed to restore MM formation, an extended subset of genes (*mamLQBIEMO)* from the large *mamAB* operon combined in a synthetic expression cassette was sufficient to induce the formation of intracellular membranes in the presence of the auxiliary *mms6*, *mamGFDC* and *mamXY* operons. Furthermore, we developed a system to trigger synchronous MM biogenesis by inducible *mamB* expression, which enabled us to dynamically track *de novo* magnetosome formation by time-lapse fluorescence microscopy and CET. This revealed that magnetosomes originate at unspecific cytoplasmic membrane locations by rapid MM invagination. Overall, MM formation involves a larger number of partially redundant protein functions, but is differently controlled than vesicle formation in eukaryotes.

## Results

### Structural characteristics of magnetosome membranes

**Fig 1:**
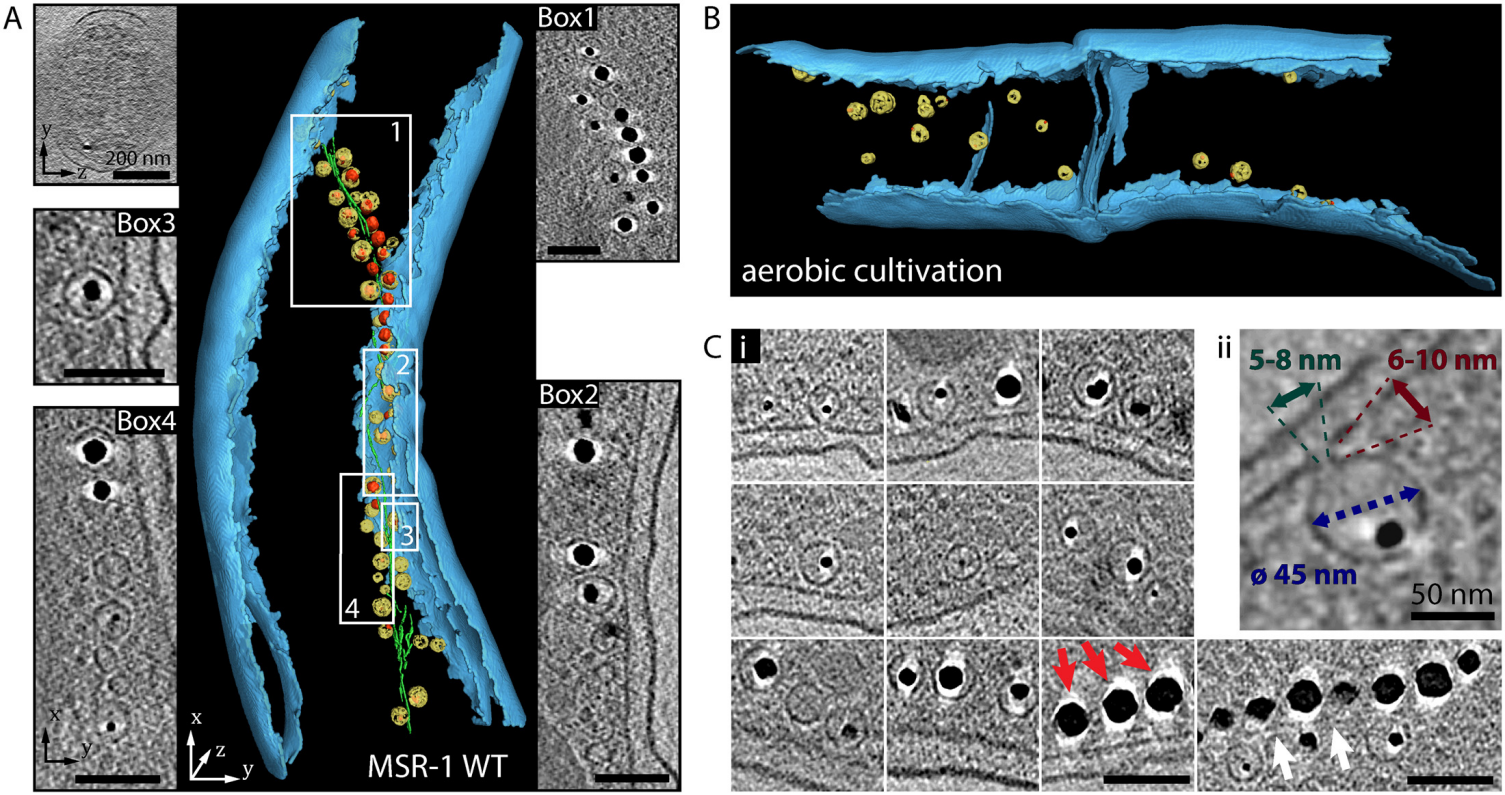
Ultrastructural analysis of magnetosome membranes from wild type. A): Segmented cryo-electron tomogram of cell with selected details from x-y slices of tomographic reconstruction (Box 1–4) and a y-z slice, illustrating information loss by the missing wedge. The outer and cytoplasmic membrane (CM) are depicted in blue, magnetosome membranes (MMs) in yellow, magnetite crystals in red and the magnetosome filament in green. Scale bars in boxes: 100 nm. (B): Segmented cryo-electron tomogram of aerobically cultivated cell that contains MMs (some with small crystal). Full tomogram is shown in S2 Video. (C): Panel with details from x-y slices of tomographic reconstructions of MSR-1 wild type cells, showing MMs that contain magnetite crystals of different sizes. The magnetosome filament is indicated by white arrows. The halo visible around magnetite crystals (red arrow shows examples) is caused by missing wedge effects and might obscure MM identification. (Cii): Section of x-y slice from tomogram showing MM that is continuous with CM and contains a small crystal. Numbers in image represent average value for all measured MM diameters (blue) (n = 289), approximate values for the annulus diameter to the periplasmic space of continuous MMs (green) and approximate values for the length of the protruding neck between the CM and MM (red). Scale bars: 100 nm.

**Fig 2:**
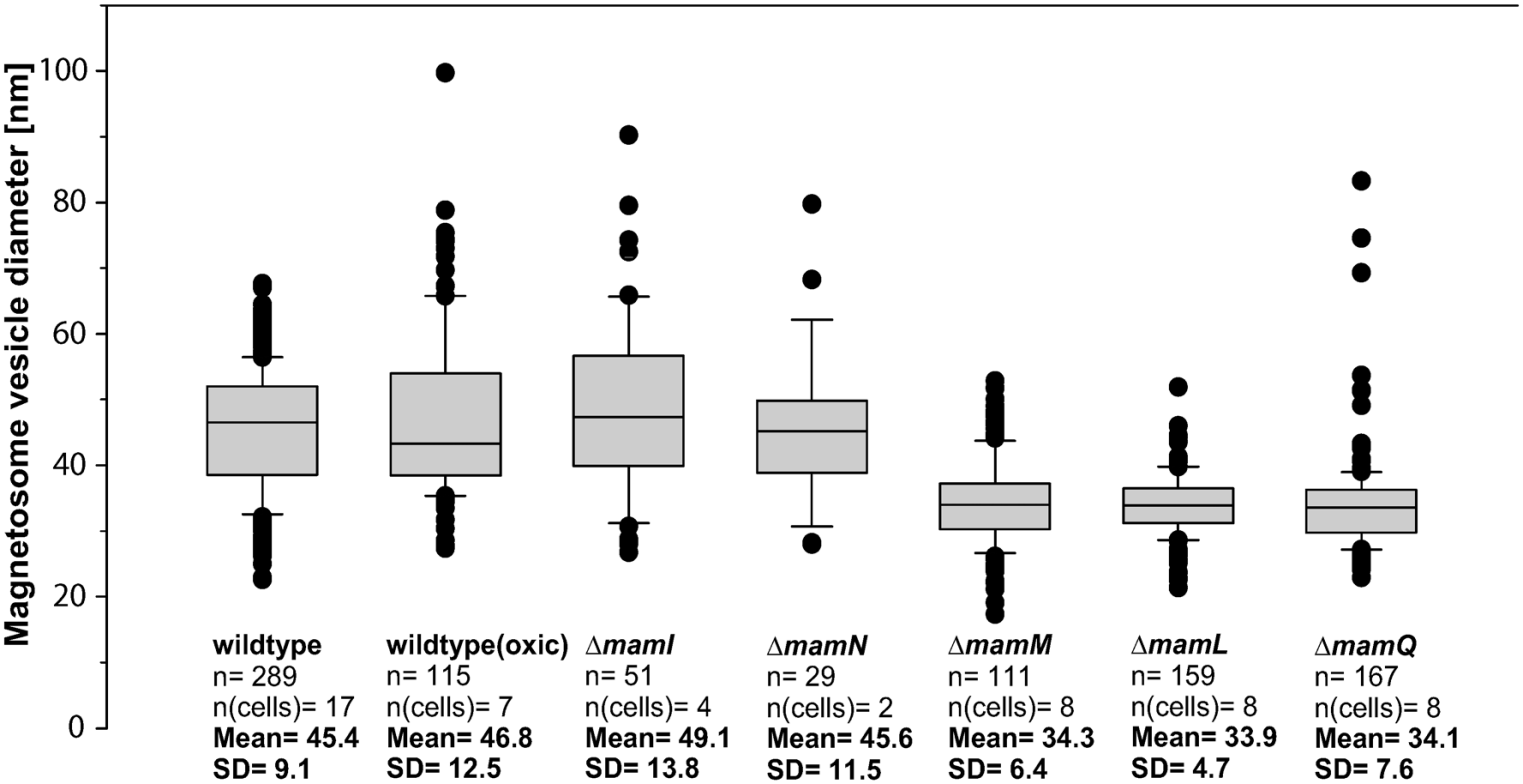
Diameters of magnetosome membranes and similar structures from wild type and several mutant strains. Diameters of magnetosome membranes (MMs) or dense MM-like structures were measured from cryo-electron tomograms. Box plots are indicating 10^th^ and 90^th^ percentiles (whiskers), 25^th^ and 75^th^ percentiles (box), median and outliers. The number of measured membranes [n] and analyzed cells [n(cells)] are indicated. The mean value and the standard deviation (SD) of the diameters are given for each strain.

To analyze the size, position and structure of regular MMs from the MSR-1 wild type, we imaged intact cells by CET (Fig 1). When cultivated under standard microoxic conditions, sizes of intracellular MMs ranged from 23 to 68 nm (mean 46 nm) (Figs 1 and 2). Partially empty MMs of similar but more variable size were also observed in aerobically cultivated non-magnetic wild type cells (Figs 1B and 2), showing that MM formation is not suppressed by conditions known to inhibit biomineralization of magnetite [21]. MMs were always found in close proximity to the cytoplasmic membrane (CM) (Fig 1C). Although the missing wedge problem of tomography allows unambiguous interpretation only in a limited area of the cell, some MMs clearly appeared as vesicles that are disconnected from the CM (S1 Video and S2 Fig). Other MMs were continuous with the CM by a protruding neck with a length of around 6–10 nm, mouthing into the periplasm by a seemingly unobstructed annulus (Fig 1Cii), whose diameter of around 5–8 nm should allow the diffusion of small molecules or even proteins between the periplasm and the MM lumen. To explore this idea, we created a strain in which enhanced green fluorescent protein (EGFP, estimated size 2.4 x 4.2 nm [22]) was directed to the periplasm by fusing the protein to a twin arginine transporter (TAT)-export signal peptide (RR) derived from a putative hydrogenase subunit of MSR-1. As indicated by an even fluorescence signal in the cell periphery, RR-EGFP was transported into the periplasmic space (S3 Fig). However, we found no enrichment of RR-EGFP at positions of the magnetosome chain and failed to detect EGFP in immunoblots of MM proteins from purified magnetosome particles (S3B and S3C Fig). Similar results were obtained when cells were cultivated in the presence of the soluble, but CM-impermeable dye 5(6)-Carboxyfluorescein (376 Da) which failed to become entrapped in regions of putative magnetosome locations (S3D Fig).

### Identification of the gene complement controlling magnetosome membrane formation

**Fig 3:**
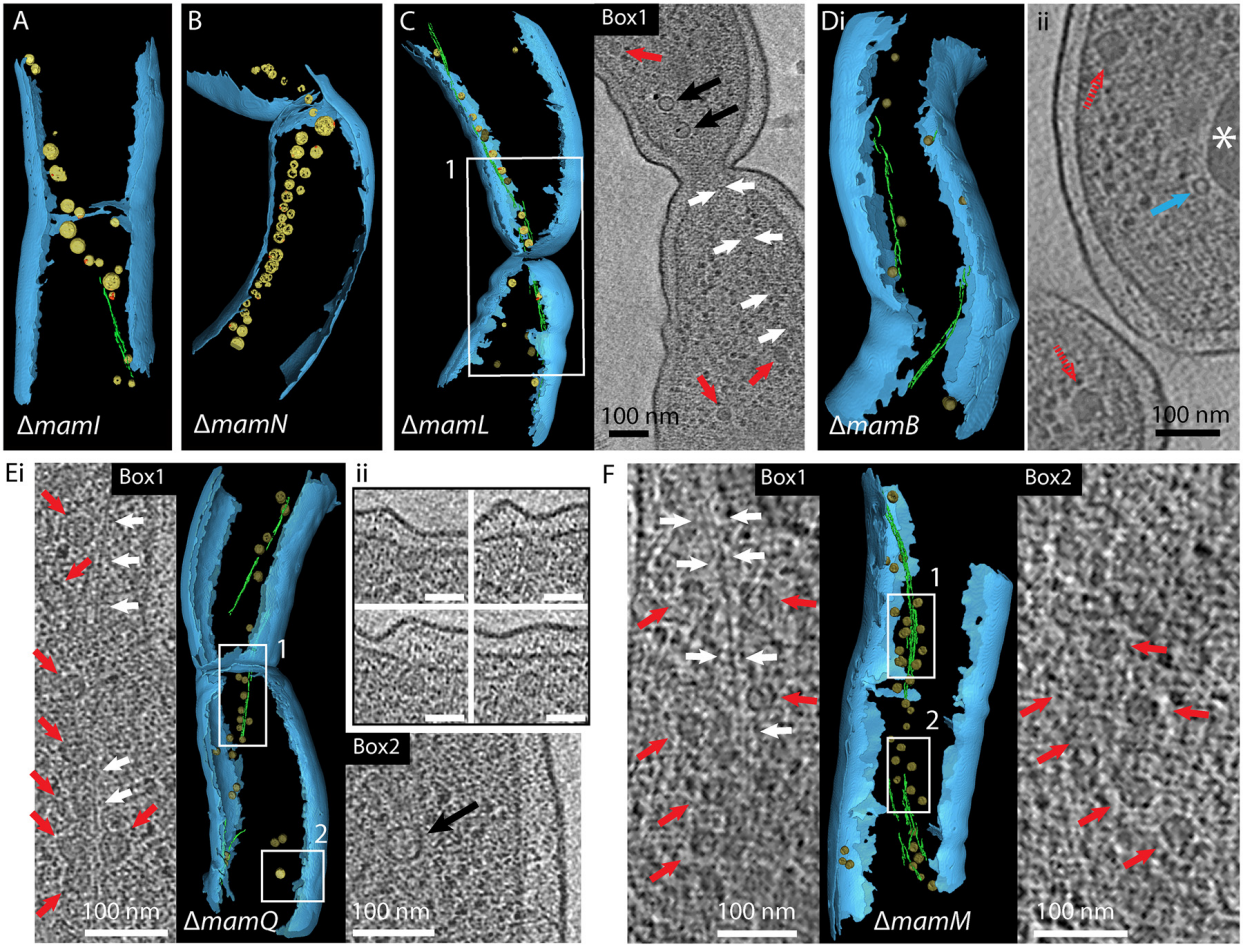
Cryo-electron tomograms of different MSR-1 mutant strains. Segmented tomograms show representative phenotypes of mutants (compare with S1 Table). The inner and outer membrane of the cells are depicted in blue, wild type-like magnetosome membranes (MMs) in yellow [black arrow in x-y slices], iron-minerals in red and the magnetosome filament in green [white arrow in x-y slices]. Distinguishable dense magnetosome membrane-like structures (DMMs) are depicted in dark yellow [red arrows in x-y slices], emphasizing the differential appearance in contrast to wild type-like MMs. Full tomograms are shown in S3–S8 Videos. (A): Δ*mamI* cell containing wild type-like MMs that partially enclose mineral particles. (B): Δ*mamN* cell containing a dense chain of empty and partially magnetite-filled wild type-like MMs. (C): Δ*mamL* cell with x-y slice detail (Box 1), showing small wild type-like MMs, partially containing crystals, and potential DMMs. (Di): Δ*mamB* cell displaying some putative isolated DMMs. (Dii): x-y slice detail of another tomogram shows putative DMMs (dashed red arrows) and a “mini-inclusion” structure (blue arrow) occasionally seen also in tomograms of the wild type and several other mutants. Asterisks marks polyhydroxyalkanoate inclusion that also occurred in all other analyzed strains. (Ei): Δ*mamQ* cell with two x-y slice details (Box 1 and Box 2). Box 1 shows filament-attached DMMs, Box 2 shows putative wild-type like MM. (Eii): x-y slice sections of another tomogram show four putative DMMs of which some appear continuous with the cytoplasmic membrane. Scale bars: 50 nm (F): Δ*mamM* cell with two x-y slice details (Box 1 and Box 2) showing filament attached DMMs.

We assessed the roles of all suspected candidate genes by thorough CET analysis of the respective mutant strains. Both Δ*mamN* and Δ*mamI* of MSR exhibited MM vesicles with a similar size, shape and chain-like alignment at midcell like in the wild type (Figs 2, 3A and 3B and S1 Table). Some, but not all vesicles contained electron dense particles as were already detected previously by TEM [15] (Fig 3A and 3B). In a recent study, Δ*mamL* cells grown under standard conditions appeared devoid of crystalline particles, but occasionally contained few tiny conspicuous electron-dense structures that were difficult to discern by conventional TEM of dried cells [15] (see S4 Fig). Indeed, careful examination by CET clearly revealed the presence of less abundant and small MM vesicles in more than half of the analyzed Δ*mamL* cells, occasionally also aligned in a chain and associated with the magnetosome filament (Figs 2 and 3C and S1 Table). Although cells displayed no detectable magnetic response (C_mag_), some vesicles contained tiny (<10 nm) electron-dense particles. If grown at lower temperatures known to enhance magnetite biomineralization [21], a weak magnetic response of Δ*mamL* cells became detectable (C_mag_ of 0.08 at 15°C) and cells indeed formed larger [mean: 17.2 ± 5.4 nm] and presumably more [14.2 ± 6.7] magnetite particles, now also clearly visible in dried cells observed by conventional bright field TEM (S4 Fig).

**Fig 4:**
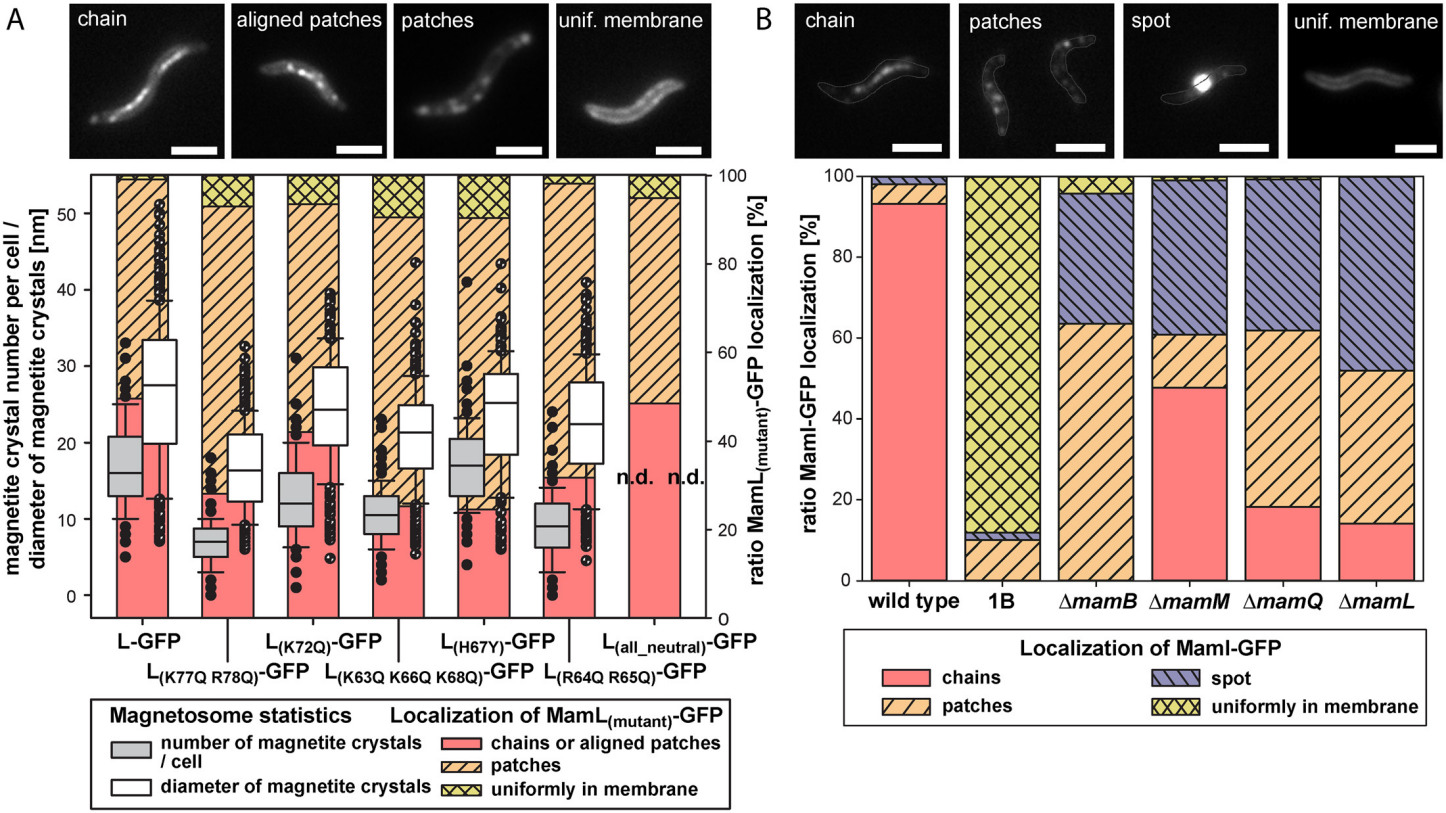
Localization of MamI-GFP in several mutants and complementation/localization assay of mutated MamL-GFP. (A) Effects of exchange of basic amino acid residues in the C-terminus of MamL, fused to EGFP by an alpha-helical linker. Quantitative analysis of magnetite crystal number/cell (grey) and magnetite crystal sizes (white) of MSR Δ*mamL* complemented with transposon-integrated P_*mamDC45*_-*mamL-egfp*, P_*mamDC45*_-*mamL*_K77Q-R78Q_*-egfp*, P_*mamDC45*_-*mamL*_K72Q_*-egfp*, P_*mamDC45*_-*mamL*_K63Q-K66Q-K68Q_*-egfp*, P_*mamDC45*_-*mamL*_H67Y_*-egfp*, P_*mamDC45*_-*mamL*_R64Q-R65Q_*-egfp* and P_*mamDC45*_-*mamL*_all neutral_*-egfp*. Box plots are indicating 10^th^ and 90^th^ percentiles (whiskers), 25^th^ and 75^th^ percentiles (box), median and outliers. Over 100 cells and 200 magnetosomes where analyzed for each strain. For quantitative analysis of MamL_(mutant)_-EGFP localization (colorful bars in background), fluorescence patterns were grouped into three classes (examples are indicated; chain and aligned patches are visualized as one class). Scale bars: 2 μm. (B) Quantitative analysis of localization of plasmid expressed P_*mamDC45*_-*mamI*-*egfp* in MSR-1, MSR 1B, Δ*mamB*, Δ*mamM*, Δ*mamQ* and Δ*mamL*. The localization patterns in individual cells were grouped into four different classes (examples are indicated; boundary of cells are outlined). More than 100 cells where analyzed for each strain. Scale bars: 2 μm.

The unexpected detection of MMs and magnetite particles in the absence of *mamL* prompted us to closer assess the function of the protein, and in particular its C-terminal domain that contains nine conspicuous basic amino acid residues that were previously implicated in MM formation [7,23]. Partial replacements by similar neutral amino acids only caused mild magnetite biomineralization defects, while neutralization of all nine residues (MamL_all neutral_) copied the severe biomineralization phenotype of the Δ*mamL* mutant (Fig 4A). Nevertheless, MamL_all neutral_-EGFP and all other GFP fused mutant versions of MamL partially localized in linear chains or aligned patches at midcell, similar to MamL_wild type_-EGFP, suggesting that the positively charged C-terminal residues are not essential for MM-tubulation or -interaction but rather participate in a process related to magnetite maturation (Fig 4A, see S1 Text for details).

Interestingly, in tomograms of Δ*mamL* we noticed vesicular structures that coexisted with smaller wild type-like vesicles but were entirely devoid of electron dense particles and had a distinct, almost uniformly dense appearance, in contrast to the light lumen enclosed by an electron-denser membrane in wild type-like vesicles (Fig 3C). However, their alignment with the linear magnetosome chain suggested that they are related to magnetosome vesicles (Fig 3C). To distinguish them from regular MM vesicles, we termed these novel structures “dense magnetosome membrane-like structures” (DMMs). Similar DMMs were even more abundant in tomograms of Δ*mamQ*: While approximately 25% of the analyzed cells were devoid of any vesicular structures in tomograms, we occasionally also detected isolated empty vesicles of rather wild type-like MM appearance (Fig 3Ei). However, large quantities (up to 50) of small, closely spaced and magnetosome filament-associated DMMs were present in 7 of the 16 analyzed Δ*mamQ* cells (Figs 3E and 2 and S1 Table). Interestingly, we also imaged nascent DMM structures apparently connected to the CM (Fig 3Eii). This, together with their association with the magnetosome filament and their compact chain arrangement, strongly suggests that these structures represent aberrant states of the MM. To gain a better understanding about the function of MamQ, we N-terminally labeled the protein with fluorescent EGFP and mCherry. The fusion proteins mainly localized in distinct but highly mobile patches in the CM and in immunoblots mCherry-MamQ showed lower abundance in purified magnetosomes. Additionally, amino acid substitution of five conserved and putative surface exposed aromatic or acidic residues that might possibly be involved in protein-protein interactions, inactivated EGFP-MamQ function in terms of magnetite formation (see S2 Text and S6 and S7 Figs for more details). These results might hint towards a role of MamQ in organizing other proteins within the CM.

Another pair of candidate genes implicated in magnetosome formation are *mamM* and *mamB* [8,19]. Again and as in Δ*mamQ*, we found 6 out of 16 analyzed cryo-electron tomograms of Δ*mamM* cells to contain small abundant filament-attached DMMs, while also sometimes a few scattered wild type-like empty MM vesicles were observed (Figs 3F and 2 and S1 Table). In stark contrast, Δ*mamB* cells were devoid of wild type-like vesicles, and only 5 of the 18 analyzed Δ*mamB* cells exhibited few isolated structures, resembling aberrant DMMs, but often lacking the characteristic association with the magnetosome filament and never aligned in coherent chains, as seen in Δ*mamQ* and Δ*mamM* (Fig 3D and S1 Table). Thus, deletion of *mamB* caused the most severe MM phenotype of all tested candidate genes.

### The magnetosome marker MamI-GFP shows chain-like localization in Δ*mamL*, Δ*mamQ*, Δ*mamM*, but not in Δ*mamB*

Next, we were interested how the variable impairments of MM formation in the mutant strains affected the localization of other magnetosome proteins. To this end, we expressed a MamI-EGFP fusion as MM marker [16,19,24] in Δ*mamL*, Δ*mamQ*, Δ*mamB*, Δ*mamM*, as well as in wild type and MSR-1B (a spontaneous mutant lacking all magnetosome-related MAI genes except the auxiliary *mamXY* operon [25]). In contrast to the confined linear chain signal prevailing in the wild type (Fig 4B), localization of the MamI-GFP signal was drastically altered in MSR-1B: the fluorescence was homogeneously distributed over the CM in most cells, indicating that MamI-GFP entirely lost specific localization in the absence of MM and most other magnetosome proteins (Fig 4B). In Δ*mamB*, no linear chain localization was visible as expected due to the absence of MM vesicle chains in this strain. However, MamI-GFP was predominantly localized in patches in the membrane or in a single spot at midcell, suggesting that the presence of other magnetosome proteins already caused MamI-GFP to accumulate in distinct foci (Fig 4B). In contrast, when expressed in Δ*mamM*, Δ*mamQ*, and Δ*mamL* backgrounds, MamI-GFP localized as linear signals of short to intermediate length in approximately 48%, 18%, and 14% of the analyzed cells, respectively, while other cells showed fluorescent patches in the CM and single strong foci (Fig 4B). The partially linear MamI-GFP fluorescence is thus consistent with the chain-organized MMs or DMMs also observed by CET in some cells of these strains, indicating that MamI is present in both types of membranes and further corroborates that DMMs are indeed magnetosome-like structures.

### Co-expression of *mamLQBIEMO* restores internal membrane formation, but not magnetite biosynthesis in the absence of other *mamAB* operon genes

**Fig 5:**
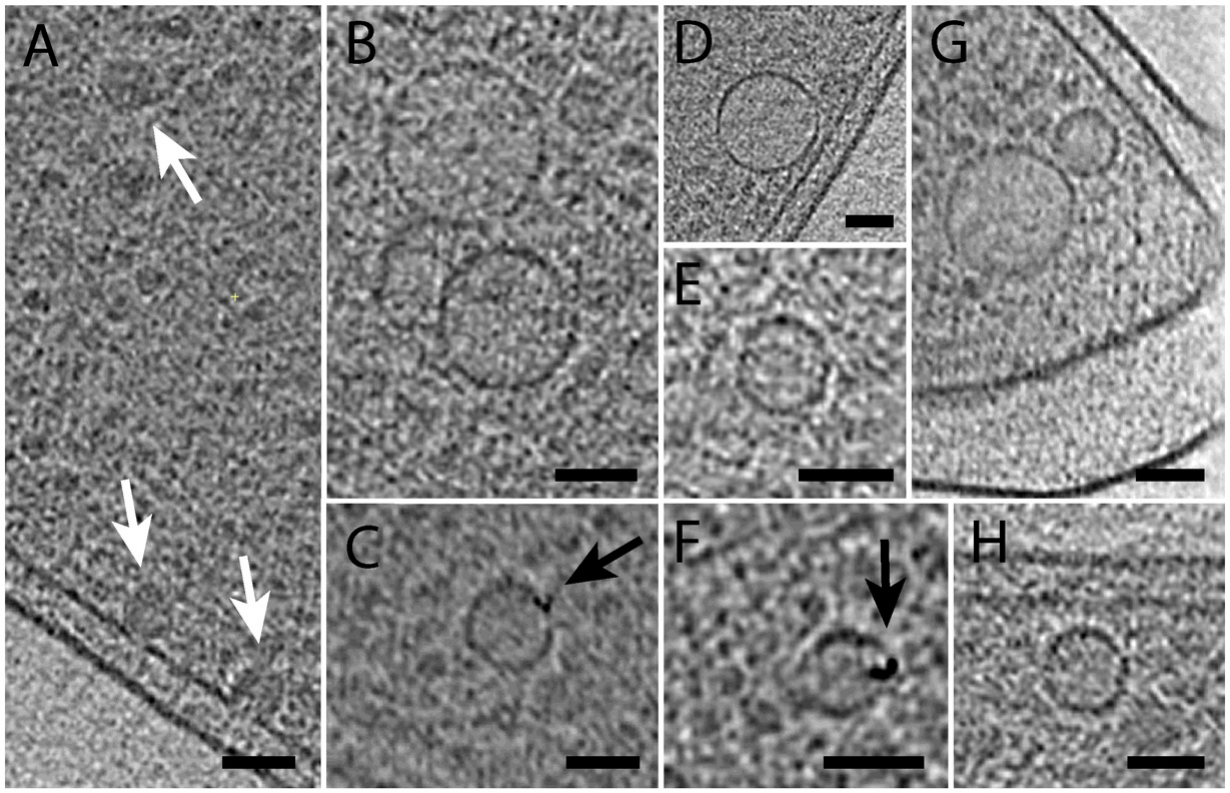
Internal membranes formed by expression of *mamQLBMEIO* in Δ*mamAB*. x-y slices from cryo-electron tomograms, showing typical magnetosome-vesicle like internal membrane structures in Δ*mamAB* P_*mamAB*_*-mamLQB-*P_*mamAB*_*-mamIEMO*. Image A shows structures reminiscent of dense magnetosome membrane-like structures (white arrows). Images B, D, and G show internal membranes that are larger than regular magnetosome membranes. Images E and H show internal membranes within the normal size range of wild type magnetosomes. Images in C and F show conspicuous electron dense inclusions (black arrow) within vesicles of wild type magnetosome size. Scale bars: 50 nm.

We found that MamL, MamQ, MamM and particularly MamB are the only proteins that play important roles in the formation of regular MMs. To test whether these genes are altogether also sufficient for MM formation in the absence of other magnetosome genes, we constructed artificial operons, first combining the native *mamAB* promoter with *mamL*, *mamQ*, *mamR*, *mamB*, and in a second version adding *mamM*. The small *mamR* gene was conveniently retained between *mamQ* and *mamB* to maintain the native gene order. While both constructs upon chromosomal integration restored magnetic responses in the single gene deletion strains Δ*mamL*, Δ*mamQ*, Δ*mamB* and Δ*mamM*, respectively, expression in MSR-1B did neither restore biomineralization of particles nor MMs as assayed by TEM and CET. A third construct (pBAM-minMAI) comprised two fully synthetic expression cassettes with the entire complement of genes individually found to be essential for magnetite biomineralization in MSR-1 [15] and each controlled by an independent copy of the P_*mamAB*_ promotor (P_*mamAB*_-*mamLQB* and P_*mamAB*_-*mamIEMO*). Chromosomal integration and expression of the construct in Δ*mamQ* and Δ*mamM* backgrounds partly restored the magnetic orientation of the strains (>60% wild type C_mag_), but not in MSR-1B or Δ*mamAB* (lacking all genes of the *mamAB* operon). However, while expression in MSR-1B also did not notably induce internal membrane formation (rare vesicular structures were detected), empty membrane vesicles became clearly visible in Δ*mamAB* cells expressing the construct (Fig 5): 11 of 15 analyzed tomograms contained at least 2–3, but up to 14 vesicular structures that were on average larger (62 ± 21 nm, up to 105 nm) than MMs of the wild type. Besides their unspecific localization along the cell body, we sometimes detected these membrane structures accumulated in close proximity to cell poles, an atypical position for magnetosomes (Fig 5G). Notably, four of all detected vesicles did contain very tiny (5–10 nm) electron dense particles of unknown identity (Fig 5C and 5F). Other structures in the cells were reminiscent of DMMs (Fig 5A). For comparison, similar single vesicular structures were only observed in 2 out of 11 analyzed cells of the Δ*mamAB* parent strain. These results indicate that the *mamLQBIEMO* genes alone are insufficient to restore magnetite biomineralization, but sufficient to induce the formation of intracellular membranes in the presence of the auxiliary *mms6*, *mamGFDC* and *mamXY* operons.

### Induction of MamB expression reveals dynamics of *de novo* magnetosome membrane formation

**Fig 6:**
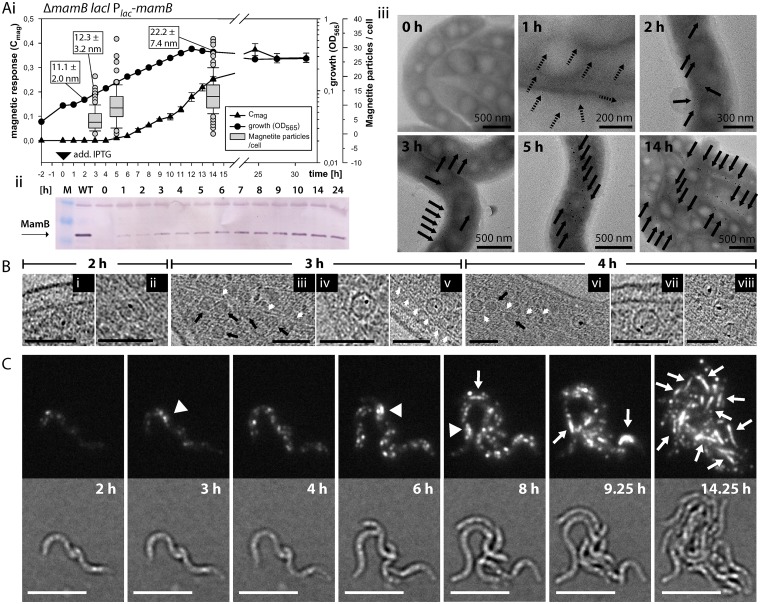
Induction of *mamB* and *mamB*-GFP expression enables *de novo* magnetosome formation. (Ai): Progression of growth (OD_565_ [circles]) and magnetic response (C_mag_ [triangles]) over time after induction of *mamB* expression with 2 mM IPTG in strain MamB_ind_. IPTG was added at time point 0 (black triangle). TEM micrographs of formaldehyde-fixed cells were utilized to determine the number of magnetite particles per cell (box plots) and average magnetite particle diameter (white boxes) at certain time points. (Aii): Western blot with immune-detection against MamB after SDS-PAGE with whole cell samples from experiment (i) taken at certain time points. The cell density of the samples was normalized based on OD_565_. (Aiii): Examples of TEM micrographs of formaldehyde-fixed cells obtained at different time points of experiment (i). Black arrows indicate the positions of single or multiple magnetite crystals (tiny particles after one hour of induction might stem from background). (B): Details of x-y slices from cryo-electron tomograms acquired with MamB_ind_ cells, plunge-frozen at a various time points after induction with 2 mM IPTG in a separate experiment. (i) and (ii): Details from two cells 2 hours post induction. (iii-v): Details from one cell 3 hours post induction. (v-viii): Details from two cells 4 hours post induction. Putative DMMs are indicated by black arrows, the magnetosome filament by white arrows. Scale bars: 100 nm. (C): 24 hours time-lapse live-cell fluorescent microscopy of induced MamB-EGFP_ind_ strain. Cells were grown at 30°C on sealed 1% agarose pads containing modified FSM medium and 3 mM IPTG. Fluorescence and corresponding bright field images from various indicated time points after induction are shown. White arrowheads indicate accumulation of fluorescent patches at midcell, while white arrows indicate linear fluorescence signals within cells. Scale bar: 2 μm.

All experiments described so far only yielded a static view on MM biogenesis. To resolve the spatiotemporal dynamics of magnetosome *de novo* formation, we designed an inducible genetic system that allowed the tuned expression of key magnetosome genes (see Materials and Methods). We first tested induction of MamL expression from the *lac* promoter in a Δ*mamL* background. As expected, this restored magnetosome formation back to wild type levels within several hours and also the proper localization of the previously mislocalized magnetosome marker protein MamC-EGFP (S8 and S9 Figs). However, as noted during the course of this study, the presence of previously undetected MM vesicles and magnetite crystals in Δ*mamL* rendered this strain inappropriate to analyze *de novo* MM formation. We therefore engineered an analogous strain for the genetic induction of *mamB* (MamB_ind_), which had emerged as the most important gene for MM formation in this study. In the absence of IPTG, no MamB expression was detectable in MamB_ind_ by immunoblots, verifying its desired tight repression (Fig 6Aii). However, 1 hour after IPTG addition, MamB expression became apparent and its levels further increased gradually over approximately the next 10 hours, after which MamB levels remained constant (Fig 6Aii). Yet, a magnetic response (C_mag_) of the cells became detectable only 5 hours post induction, but further steadily increased until the end of the experiment (Fig 6Ai). The cells were devoid of any electron-dense particles before induction, but few isolated and several concatenated magnetite particles with an average diameter of around 11 nm became discernable by TEM within few cells already after 2 hours (Fig 6Aiii). The crystal size was further increased 3 hours after induction, and cells on average contained 5 (up to 20) magnetite particles, while only very few cells remained completely devoid of crystals (Fig 6Ai and 6Aiii). Whenever multiple particles were visible, they predominantly already assembled as a loosely spaced chain at midcell (Fig 6Aiii), even when still in their superparamagnetic size range below 15–20 nm [26]. Although after 14 hours the cells on average contained still fewer (14, max. 33) and smaller (around 22 nm) magnetosomes than wild type cells (Fig 6Ai and 6Aiii), the slow *de novo* development of chain-aligned magnetosomes thus rendered the strain particularly useful for resolving the dynamics of MM formation over time. We pursued this in the next experiment in which cells were plunge-frozen and analyzed by CET at distinct time points after induction: Wild-type like MM vesicles were absent from the non-induced strain, but single crystal containing MM vesicles became visible at least 2 hours post induction (Fig 6Bi and 6Bii), along with magnetosome filament-associated DMMs. 3 hours post induction, the numbers of MM vesicles had increased and few crystal-containing, filament-associated and loosely aligned MMs became visible both at the inner and outer curvature of the helical cells. They were mostly found in the recorded areas nearby the future cell division sites, sometimes adjacent or in several 100 nm distance from each other. Furthermore, both closely chain-aligned DMMs and independent single crystal-containing magnetosomes were found within the same cells (Fig 6Biii–6Bv). 4 hours post induction, two cells exhibited short and coherent magnetosome assemblies (up to three vesicles) with immature crystals (Fig 6Bviii). One cell contained both DMMs and wild type-like MM vesicles associated with the same visible section of the magnetosome filament (Fig 6Bvi), while another cell contained >10 chain-aligned DMMs. Altogether, the CET results suggested that wild-type MM formation after induction of *mamB* expression proceeded slowly and gradually, rather than by the simultaneous formation of large numbers of MMs. Notably, despite very careful examination, we failed to identify any structures resembling intermediate stages of early MM development (e.g. crystal bearing membrane transformation stages other than spherical invaginations) in any of the 23 analyzed tomograms from any time point (1, 2, 3 or 4 hours post induction). This indicates that if potential intermediary structures exist, they must likely be very transient and differentiate into spherical MMs before biomineralization is initiated.

Because of the low time resolution of CET sampling, we also attempted to track magnetosome formation in living cells by time-lapse fluorescence microscopy in a strain in which an inducible MamB was fused to EGFP (MamB-EGFP_ind_.) This fusion was previously demonstrated to become recruited into the MM [8] and shows a linear localization within cells that form magnetosome chains (see S7A Fig). As MamB_ind_, MamB-EGFP_ind_ also developed magnetosomes after induction, as determined by a C_mag_ of approximately 0.3 after over-night induction with IPTG. Using an improved protocol (see Material and Methods), cells could be imaged for more than 24 hours and for at least up to 6 consecutive divisions in a single experiment (S10 Fig and S9 and S10 Videos). Faint fluorescence signals became visible approximately 1 hour post induction. In dividing cells, the fluorescence intensity then steadily increased over approximately the next 10 hours (≙ 2–3 divisions). During the first hours, the signal was mainly localized in multiple punctuate foci at unspecific positions within the cells, but in few frames was predominantly accumulated at midcell (Fig 6C). Approximately 8 to 9 hours post induction, the fluorescence signal developed into a linear localization at or close to midcell, co-existing with single foci at unspecific positions (Fig 6C). This linear localization then persisted, became elongated and segregated in many of the dividing cells, and could still be observed in some cells after the GFP signal gradually began to fade after 14–15 hours (≙ 3–4 divisions) post induction (Fig 6C and S10 Fig). In corroboration of the CET results, our observations indicate that newly synthesized MamB-GFP first becomes localized all over the cell body into distinct foci at the CM, from which MM formation is then orchestrated. The fluorescent foci most likely represent MamB-GFP enriched magnetosome protein clusters or single magnetosomes, which then become recruited into coherent magnetosome chains over time.

## Discussion

### MamB is the most important determinant, but not sufficient for magnetosome membrane formation

Previously, various proteins (MamB, I, L, Q, Y) were assumed to be essential for MM formation and/or to actively participate in membrane remodeling [19,20,27,28]. However, we found that *mamI* is not required for the formation of wild type-like MMs in MSR-1. Contrary to previous assumptions, also single deletions of *mamQ* and *mamL* continued to form MMs or similar aberrant structures. While the requirement of *mamM* for MM formation was already challenged [8], its deletion also caused the formation of aberrant internal membranes. Most unexpectedly, we found no single candidate protein to be absolutely essential for MM formation, since even in the Δ*mamB* mutant strain, which exhibited the most severe MM phenotype, rare aberrant membrane structures could be detected in few cells by careful CET analysis. However, since loss of MamB abrogated the formation of abundant concatenated internal membranes, it emerged as the most important factor for MM biogenesis.

The severe impairments in magnetite crystallization in the absence of MamL suggest that besides its participation in MM formation, this protein is primarily involved in the maturation of magnetite crystals, consistent with its universal presence in all magnetite-producing MTB, but absence from greigite producers [29]. The positively charged carboxy-terminus of MamL seems to be highly important for this function. MamL could either act directly on magnetite biosynthesis, or alternatively might be involved in the organization and recruitment of other magnetite maturation proteins, as suggested by the variable degrees of MamI-GFP and MamC-GFP mislocalization in Δ*mamL*, and the magnetosome recruitment of the MamC-GFP upon re-induction of MamL expression.

While fluorescent fusions of other analyzed Mam proteins predominantly localized to the MM, mCherry-MamQ, was mainly localized in the CM. The putative surface exposed, highly conserved acidic and aromatic amino acid residues that we found to be important for the function of MamQ might be involved in direct electrostatic or stacking interactions with other magnetosome proteins. Thereby, MamQ might participate in magnetosome formation by acting as a hub in the CM for the early organization of magnetosome proteins prior to membrane invagination.

Expression of *mamB* together with all other genes (*mamQ*, *L* and *M*) affecting MM formation in our study was not sufficient to restore MM biogenesis in absence of the other 21 genes from the *mamAB*, *mms6* and *mamGFDC* operons. Only co-expression of the synthetic *mamLQBIEMO* construct in a Δ*mamAB* strain restored the formation of few intracellular membranes reminiscent of MMs. However, as indicated by the lack of similarly abundant structures upon expression in the MSR-1B background, intracellular membrane formation is supported by the presence of additional genes from the *mms6*, *mamGFDC and mamXY* operons. This is unexpected, since deletion of these operons, alone or in combination, did not affect MM formation in previous studies, and the *mamAB* operon alone was sufficient to sustain rudimentary magnetosome formation in both MSR-1 and AMB-1 [30,31]. Altogether, this suggests that several MM proteins have redundant (i.e. apart from their specific functions e.g. in magnetite biomineralization) and cumulative functions in MM biogenesis, and factors outside the *mamAB* operon might be required for MM formation depending on the genetic context.

Our results are in clear contrast to observations in AMB-1, where *mamI*, *-L*, *-Q and mamB* were found to be essential for MM formation, as assayed by cryo-ultramicrotomy/TEM [19]. However, similar to our findings, the combined expression of *mamILBQ* in this strain also proved insufficient to restore MM formation in the absence of other *mamAB* operon genes [19]. Despite the high conservation of their major MAI genes, and apart from the possibility that rudimentary MM-structures might have escaped detection due to technical differences in the other study, these findings hint towards discrepancies in the magnetosome formation processes between the two closely related organisms.

### Aberrant DMMs are putative precursors of regular magnetosome membranes

Empty and smaller magnetosome-like vesicles with electron-dense lumen (DMMs) became abundant upon re-induction of MM biogenesis and often outnumbered wild type-like MMs. DMMs were also found in high numbers in cells of Δ*mamM* and Δ*mamQ* and represent a previously unidentified, but distinct intracellular structure. Their association with the magnetosome filament, their apparent origin by invagination from the cytoplasmic membrane and the partial linear co-localization of the magnetosome marker MamI-GFP in mutants exhibiting chains of DMMs suggests that these structures in fact represent immature or defective MMs. The formation of DMMs could be explained by two different scenarios: i) They might just represent aberrant invaginations that are smaller due to the lower incorporated amounts of proteins, possibly caused either by the absence of early landmark proteins such MamQ and MamM, or following the artificially slow re-induction of a single key protein. ii) DMMs might be precursors that accumulate due to delayed MM biosynthesis (upon *mamB* induction) but eventually will convert into regular MMs, or became stalled at early stages in the mutants. The absence of early key proteins might prevent hierarchical recruitment of additional proteins downstream and thus, inhibit further development. If DMMs represent intermediate stages of MM biogenesis, they should transiently also occur in wild type cells. Indeed, conspicuous structures coexisting with regular magnetosomes in some cells (e.g. see S1 Video, ends of chain [right] and close to regular magnetosomes [left]) might be identical to DMMs.

### Dynamics of magnetosome membrane formation

Since their first visualization, it has remained unclear whether MMs remain permanently continuous with the cytoplasmic membrane (CM) from which they originate, or if they become eventually pinched off, thus developing into vesicles discontinuous with the CM [5,6]. In contrast to a previous study of AMB-1 in which the vast majority of MM appeared connected to the CM [6], we here identified MMs that although still in close vicinity, were clearly discontinuous with the CM, thus confirming previous observations from MSR-1 [32,33]. Similarly, a recent CET study of the magnetotactic Alphaproteobacterium *Magnetovibrio blakemorii* also failed to reveal connections of MMs with the cytoplasmic membrane [34]. Although the lumen of invaginating MMs, i. e. those in *statu nascendi*, might transiently form a continuum with the periplasm, our results suggest that the molecular exchange between the two compartments is tightly regulated or obstructed by a physical barrier.

Synchronous genetic induction enabled us to track *de novo* magnetosome biogenesis with unprecedented time resolution by electron and live-cell fluorescence microscopy. Soon after induction, MamB-GFP formed patches at the CM and later linear signals within the cells. The early punctuate fluorescent signals might represent local protein clusters in the CM or early magnetosomes. This coincided with the appearance of single nascent magnetosomes in tomograms. As already speculated from previous iron-induction experiments [5], magnetosomes therefore do not originate only from specific locations within the cell, but appear along the entire length, before they are concatenated into closely spaced chains. Single nascent magnetite-containing magnetosomes were often already attached to the MamK magnetosome filament, indicating that the cytoskeletal structure becomes connected with the newly developing MM immediately, or even plays an assisting role in orchestrating of early magnetosome formation. Later, when magnetite crystals were still in the superparamagnetic size range, crystal-containing MMs in tomograms were still found isolated or in very short chains at different positions within dividing cells. According to results from TEM and fluorescent microscopy, magnetosomes then became organized in dense chains at mid-cell, presumably by the action of the magnetosome filament.

In a very recent study published during revision of this manuscript, MM formation was genetically induced via the *mamQ* gene in the related AMB-1 [35]. The results of this study also suggested that the machinery required for magnetosome membrane formation is distributed at multiple sites throughout the cytoplasmic membrane. Nascent magnetosome membranes became first organized into linear, but discontinuous long-range aligned assemblies, after which the gaps between adjacent magnetosomes were closed by a mechanism dependent on the MamK magnetosome filament [35].

### Magnetosome membrane biogenesis is a multi-determined process

**Fig 7:**
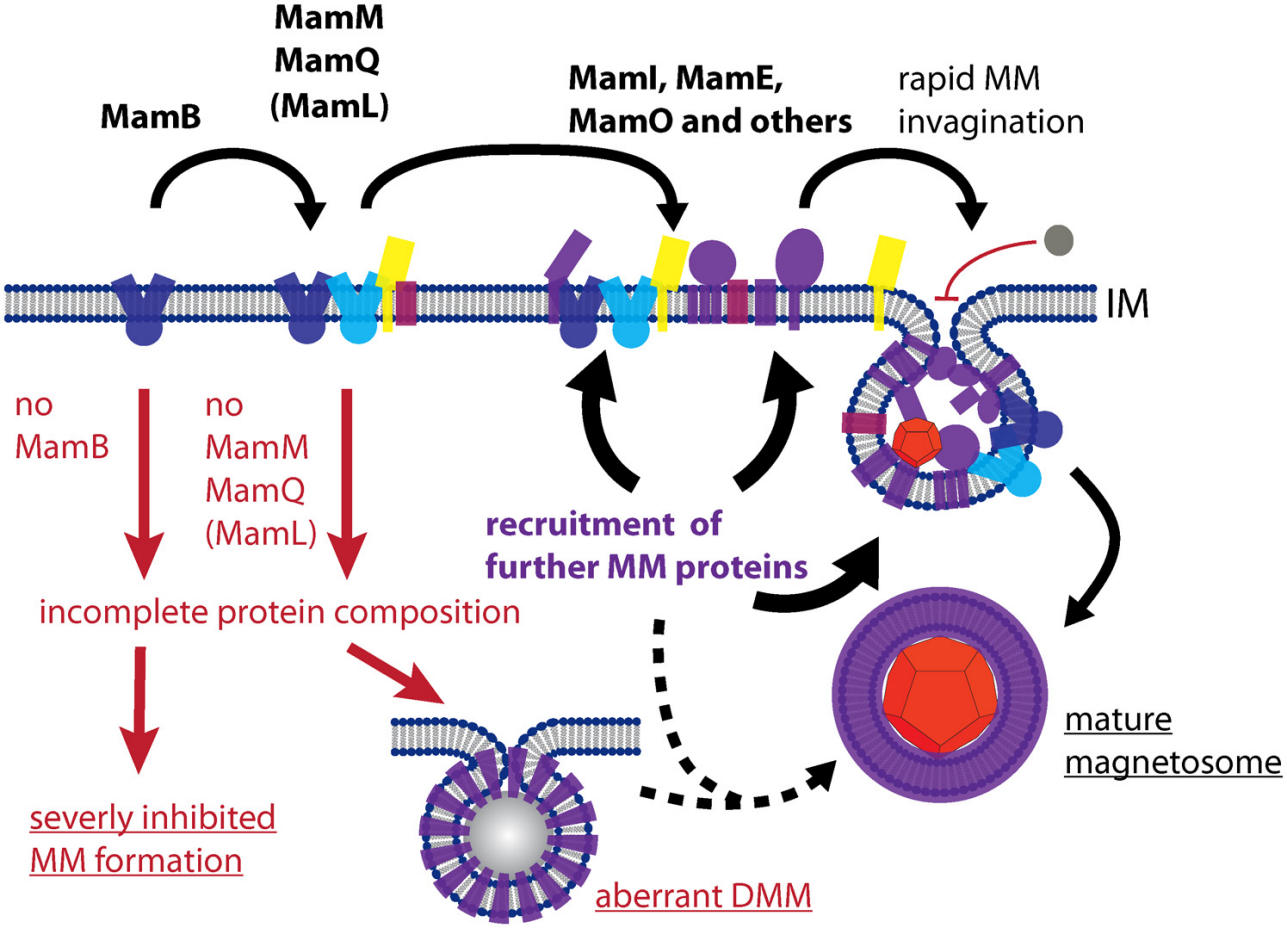
Hypothetical model for magnetosome membrane formation. The model suggests that magnetosome membrane proteins (colorful shapes) are recruited to certain sites of the cytoplasmic membrane in a hierarchical manner, with the key proteins MamB, MamM, MamQ and MamL (labeled in shades of blue, yellow and red) acting as nucleating factors, which is followed by recruitment of MamI, MamE, MamO and other magnetosome proteins. Since MamB was found most important for magnetosome membrane (MM) formation, it might act as the initial landmark protein to prime complex formation at certain sited within the cytoplasmic membrane. After a critical size and composition of the multi-protein assembly is reached, the formed lipid-protein complex induces rapid invagination to form the magnetosome lumen. Diffusion from the periplasm into this lumen is blocked. Later, several further magnetosome proteins might become recruited into the MM, which eventually becomes detached to form magnetosome vesicles. The absence of MamB strongly inhibits MM formation, while the absence of either MamM, MamQ or MamL might cause a disturbed protein composition, which leads to the formation of aberrant dense magnetosome membrane-like structures (DMMs) that lack magnetite crystals or blocks magnetosome formation at an immature state.

As shown by several studies, the lack of certain magnetosome proteins can affect the proper localization of others. For instance, MamC mislocalized upon deletion of *mamQ*, *mamA*, *mamM* [8,15], and, as presented here, also *mamL*. Similarly, MamA, MamI and MamC mislocalized upon deletion of *mamE* [16,19], which indicates a hierarchical recruitment of proteins to the MM. Based on our and previous findings, we delineate a hypothetical model for MM biogenesis: The analyzed key proteins MamB, MamM, MamQ and MamL mark the beginning of a recruitment cascade and are required to position a network of additional magnetosome proteins, including MamI, MamE and MamO (Fig 7). In turn, recruitment of further magnetosome proteins and oligomerization into high molecular weight complexes may introduce curvature into the cytoplasmic membrane (Fig 7), as was already previously speculated by Nudelman *et al*. [36]. This could be similar to ICM formation in *Rhodobacter sphaeroides* where simulations predicted that regular insertion of the curved multi-protein RC-LH1-PufX photosynthetic “core” complex and arrays of LH2 complexes into a model membrane can cause membrane curvature, tubulation and invagination [37,38]. Similarly, the compact assembly of magnetosome proteins in a pre-complex within the CM might lead to membrane invagination. In fact, previous studies with bacteria that naturally lack ICM structures (such as *E*. *coli*) already suggested that the formation of internal membranes can be induced by the overexpression of unspecific membrane proteins [39,40].

In eukaryotes, the formation of membrane vesicles is mediated by specific proteins that either form coats, scaffolds or insert into the membrane to create local curvature [41–44]. However, homologs of well-studied factors controlling vesicle formation in eukaryotes are absent from MTB and other prokaryotes. Our findings suggest that MM formation proceeds by the combined action of the membrane-integral core factors MamLQBIEMO and several other magnetosome proteins that are partly redundant and not all individually essential for the formation of wildtype-like MMs. Thus, the mechanisms of internal membrane formation in bacteria appear to be differently controlled than in eukaryotic cells. In summary, we present the so far most comprehensive ultrastructural analysis of the complex magnetosome organelle and identify the genetic determinants involved in the initial steps of its biogenesis. Understanding the assembly of magnetosomes provides the conceptual framework for investigating the biogenesis of other bacterial organelles and for constructing synthetic organelles for bioengineering applications [45].

## Material and Methods

### Bacterial strains, plasmids, culture conditions and C_mag_ measurement

Bacterial strains and plasmids used in this study are listed in S2 and S3 Tables. *E*. *coli* strains were cultivated in lysogeny broth (LB) medium. When necessary, kanamycin (km) was added to 25 μg mL^-1^. *E*. *coli* BW29427 and WM3064 cultures were supported with 1 mM DL-α,ε-diaminopimelic acid (DAP). Media were solidified by addition of 1.5% (w/v) agar. Unless otherwise stated, *M*. *gryphiswaldense* cultures were grown at 30°C under microoxic conditions (1% O_2_) in modified flask standard medium (FSM) [46]. When appropriate, km was added to 5 μg mL^-1^. Optical density (OD) and magnetic response (C_mag_) of exponentially growing cultures were measured photometrically at 565 nm as described previously [47]. Conjugation of plasmids were performed essentially as described earlier [48,49]. For Tn7 transposon containing plasmids, a triparental mating conjugation was applied using *E*. *coli* WM3064 strains harboring the respective plasmid and pT18mob2PmamDC-TnsAD as helper plasmid for transposase expression. In-frame markerless chromosomal gene fusions and deletions were created as earlier described [50].

### Molecular and genetic techniques

Oligonucleotides (S4 Table) were purchased from Sigma-Aldrich. Plasmids (S3 Table) were constructed by standard recombinant techniques using enzymes from Life Technologies and Agilent Technologies (see S3 Text) and confirmed by sequencing. Sequencing was accomplished using BigDye terminator v3.1 chemistry on an ABI 3700 capillary sequencer (Applied Biosystems).

### Induction experiments

Strains for *mamL*, *mamB* and *mamB-GFP* induction were created by site-specific, Tn7 transposon-mediated chromosomal integration of P_*lac*_ controlled genes from plasmids pOR118, pOR160 and pOR169, respectively (see S3 Text). For gene induction experiments, MSR-1 strains were passaged in sealed 1% oxygen gas-flushed 500 mL or 1L bottles with 100–300 mL FSM medium containing 8 mM NaNO_3_. A 30°C overnight culture was inoculated into a fresh bottle with starting OD_565_ of 0.02 to 0.05 (t = -2 h) and cultivated under mild shaking at 30°C. 2 h after inoculation, gene expression was induced by addition of 2 mM IPTG (t = 0). 2 mL samples were taken at certain time points and immediately fixed with formaldehyde for TEM analysis and OD_565_ and C_mag_ determination. For protein expression analysis, 10 mL culture samples were immediately pelleted at 4°C and stored on ice. Samples were resuspended in ice-cold Tris-HCl buffer (pH 7.4) to a final OD_565_ of 10 and frozen at -20°C. SDS-PAGE and Western blot were performed as previously described [8]. Prior to SDS-PAGE, samples were supplemented with electrophoresis sample buffer and incubated at 60°C for 10 minutes. For cryo-electron tomography preparations, a separate induction experiment was analogously conducted and cell samples were plunge-frozen on Quantifoil holey carbon molybdenum grids as previously described [51]. Less than 10 minutes passed in between sampling and plunge-freezing.

### Fluorescence microscopy and time lapse microscopy experiments

For epi-fluorescence microscopy, 3 μl samples of *M*. *gryphiswaldense* over-night cultures were immobilized on 1% (w/v) agarose pads with FSM medium salts. The samples were imaged with an Olympus BX81 microscope equipped with a 100×UPLSAPO100XO 1.4NA objective and an Orca-ER camera (Hamamatsu) and appropriate filer sets using Olympus Xcellence software. Alternatively, a DeltaVision Elite microscope (GE Healthcare), equipped with InsightSSI Illumination System, 100×Super-Plan-Apo 1.4NA objective, and a CoolSnap HQ2 CCD camera was used. Here, image acquisition was performed with SoftWoRx Suite 2.0. All samples were recorded in Z-stacks with 300–1000 ms exposure time per image. Images were processed with ImageJ 1.48s (http://imagej.nih.gov/ij/) using Fiji package (http://fiji.sc/).

For time lapse microscopy with Δ*mamB* P_*lac*_ *mamB-egfp*, an exponential growing culture was induced with 2 mM IPTG. A 3 μl sample was immobilized on 1% (w/v) agarose pads containing all FSM medium components, but reduced peptone (1 g/L) and increased NaNO_3_ (8 mM) concentrations and additional 3 mM IPTG. The pad was overlaid with a coverslip and sealed with hot liquid paraffin under constant 1% O_2_/99% N_2_ gas stream. The sample was transferred to 30°C preheated DeltaVision Elite microscope and imaged for 24 hours in 15 minute intervals (bright field and GFP fluorescence with 500 ms 32% exposure power using 475/28 nm excitation and 525/50 nm emission filter). Imaging started approximately 15 min post induction (this time point is referred as 0). Focus was maintained using implemented laser-based hardware autofocus. Lateral shifts were corrected with ImageJ 1.48s, using StackReg plugin of Fiji package (http://bigwww.epfl.ch/thevenaz/stackreg/).

### Transmission electron microscopy

For conventional TEM analysis, unstained and formaldehyde-fixed (0.075% w/v) cells were absorbed on carbon coated copper grids. Bright field TEM was performed on a FEI CM200 transmission electron microscope using an accelerating voltage of 160 kV. Images were captured with an Eagle 4k CCD camera using EMMenu 4.0 (Tietz). For data analysis and measurements, the software ImageJ 1.48s was used.

### Cryo-electron microscopy, tomogram reconstruction and analysis

Sample preparation and data acquisition were essentially performed as previously described [51]. A 300 kV FEI Tecnai F30 Polara, equipped with Gatan Post-Column Energy Filter and either 2 x 2 k Multiscan CCD Camera (Gatan) or 3838 x 3710 Direct Detector Device (DDD) K2 summit (Gatan) operated in counting and dose-fractionation mode was used for imaging. Images were recorded at nominal -5 μm to -8 μm defocus. The object pixel size was either 0.81, 0.71 (CCD) or 0.52 nm (DDD). Prior to microscopy, samples were plunge-frozen on holey carbon molybdenum grids. Increased blotting times often caused slight flattening of the cells.

Three-dimensional reconstructions from tilt series were performed with the weighted back-projection method using the TOM toolbox [52], creating 2-times binned volumes. For alignment purposes prior to reconstruction, automated fiducial tracking was frequently performed using eTomo (IMOD 4.7) [53].

Vesicle diameters were measured with ImageJ 1.48s. Segmentation of tomograms was performed using Amira software version 5.6.0 (FEI). Outer and inner cell membranes were beforehand automatically segmented using TomoSegMemTV [54].

Further experimental procedures are found in S3 Text.

## Supporting Information

S1 Text Experimental details of amino acid substitutions within MamL. (DOCX)

S2 Text Experimental details of amino acid substitutions within MamQ. (DOCX)

S3 Text Supplemental experimental procedures. (DOCX)

S4 Text References cited in supporting information. (DOCX)

S1 Fig Molecular organization of the most important genes associated with magnetosome biogenesis within the genomic magnetosome island of *M*. *gryphiswaldense*. The *mamAB* operon comprises 17 genes (from *mamH* to *mamU*), the *mamGFDC operon* (from *mamG* to *mamC*), the *mms6* operon (*mms6*, *mmsF*, *mms36* and *mms48*) and the *mamXY* operon (*mamY*, *mamX*, *mamZ* and *ftsZm*) each comprise 4 relevant genes. The extend of the regions separating the individual operons is indicated in kilobase pairs (kb).
(TIF)

S2 Fig Visualization of a magnetosome membrane vesicle clearly detached from the cytoplasmic membrane. Image slices (x-y; x-z; z-y) from cryo-electron tomogram of MSR-1 wildtype. The same magnetite-containing magnetosome membrane vesicle is indicated in all three image slices by white arrows. The vesicle resides within some distance and is clearly disconnected from the cytoplasmic membrane (CM). Outer membrane (OM) is indicated. Scale bar: 100 nm.
(TIF)

S3 Fig Periplasmic GFP and 5(6) Carboxyfluorescein do not become entrapped in magnetosome membrane compartments. The Twin Arginine Translocation (TAT) signal peptide (RR) of MSR-1 protein MGR0500 was fused to EGFP and the construct expressed in *E*. *coli* strain BW29427 and MSR-1. Fluorescent micrographs show that the modified EGFP was efficiently translocated into the periplasmic space of (A) *E*. *coli* and (B) MSR-1. No linear signal was detected within MSR-1, indicating lack of diffusion and entrapment of the protein in the MM. Left: green channel, middle: 3D-deconvoluted representation, right (in B): DIC channel. (C): Western blot with purified and protein concentration normalized fractions from MSR-1 expressing EGFP (lanes 1–3) or RR-EGFP (lanes 4–6). MM protein fraction (lane 1 and 4), total soluble protein fraction (lane 2 and 5) and total cellular membrane protein fraction (lane 3 and 6). Immunodetection was performed with GFP Antibody. Arrows indicate a putative signal for GFP and RR-GFP. The RR-signal cleavage after translocation of the protein to the periplasm can be observed in the blot. (D): Assay to determine 5(6) Carboxyfluorescein (FAM)-diffusion into MM. MSR-1 was cultivated over-night in FSM medium supplemented with 1 mM 5(6) FAM. Cells were either 3x washed in 1 volume of PBS or previously chemically fixed by addition of 0.075% formaldehyde and 5 mg/mL BSA for 15 min before washing. Left micrographs shows fixed cells that are fluorescent, indicating enclosure of 5(6) FAM, right micrograph shows unfixed cells that are non-fluorescent.
(TIF)

S4 Fig Representative TEM micrograph of MSR Δ*mamL* cultivated at 30°C and 15°C. (A): Micrograph of Δ*mamL* cell, cultivated at 30°C (under standard conditions). Inlet shows indicated area in higher magnification. Arrows indicate position of (putative) tiny magnetite particles (B): Micrograph of Δ*mamL* cell, cultivation at 15°C. Arrows indicate position of magnetite particles.
(TIF)

S5 Fig Alignment of MamL and MamL-like proteins from various magnetotactic bacteria. Basic amino acids are indicated in purple and are enriched in C-terminal regions.
(TIF)

S6 Fig Alignment of MamQ and LemA proteins from various magnetotactic bacteria and *Thermotoga maritima*. The MTB-specific stretch and the analyzed point mutated residues from this study are indicated.
(TIF)

S7 Fig Predicted structure of MamQ and localization of fluorescently labeled protein. (A): Representative fluorescence micrographs of MSR-1 cells (Ai): overexpressing P_*mamDC45*_-*mamQ*-*egfp* and (Aii): expressing chromosomal in-frame allelic replacements of *mamB*::*mamB*-GFP and *mamQ*::*mCherry-mamQ*. From left to right: DIC channel, green fluorescent channel, red fluorescent channel. Scale bars: 2 μm (Bi): Western blot with separated and concentration-normalized fractions obtained from *mamQ*::*mCherry-mamQ* cell lysate. Total soluble protein fraction (lane 1), total non-magnetic membrane protein fraction (lane 2) and magnetosome membrane protein fraction (lane 3). Primary immuno-detection was performed with MCherry antibody. (Bii): Quantitative analysis of magnetosome diameter (left) and magnetosome number (right) of *mamQ*::*mCherry-mamQ* and wild type. Box plots are indicating 10^th^ and 90^th^ percentiles (whiskers), 25^th^ and 75^th^ percentiles (box), median and outliers. Over 500 magnetosomes and 100 cells where analyzed, each. (C): Fluorescence micrographs of non-magnetic MSR-1 cells expressing in-frame chromosomal replacements of (i) *mamQ*::*egfp-mamQ*_Y241A F242A_,(ii) *mamQ*::*egfp-mamQ*_E179A_, (iii) *mamQ*::*egfp-mamQ*_Y181A_, (iv) *mamQ*::*egfp-mamQ*_E111A_ and (v) *mamQ*::*egfp-mamQ*_E111A E179A Y181A_. Scale bars: 2 μm. (D): Different views on the model of MamQ_MSR-1_ tertiary structure. The protein structure of the soluble part of MamQ (using amino acids 70–246) was modelled with SWISS-MODEL and the experimentally determined 2.28 Å resolved crystal structure of LemA_*T*.*maritima*_ [PDB ID 2ETD] as template (GMQE = 0.30, QMEAN = -6.80). Only the backbone of the structure is visualized. Putative alpha-helical regions are depicted in purple, the side chains of the mutated amino acids in this study are depicted in yellow, represented in stick and ball model and indicated by an arrow if visible in the view. They are either localized in predicted loop or flexible regions. If visible, also the N and C-termini of the modeled protein structure are indicated. The predicted trans-membrane domain of MamQ continues at the indicated N-terminus.
(TIF)

S8 Fig Induction of *mamL* expression increases magnetosome size and C_mag_ to wild type-like levels. Induction of *mamL* expression in Δ*mamL* P_*lac*_-*mamL*. In the absence of the inducer IPTG, the strain failed to exhibit a magnetic response (C_mag_ = 0) when cultivated at 30°C. Upon addition of IPTG, a gradual restoration of C_mag_ and magnetosome size and number was detected and wild type-like levels were reached after over-night incubation. (A): Progression of growth (OD_565_, circles) and magnetic response (C_mag_, triangles) over time after induction of *mamL* expression with 2mM IPTG in Δ*mamL* P_*lac*_-*mamL*. IPTG was added at time point 0 (black triangle). (B) TEM micrographs showing magnetite crystal morphology in cell from experiment (A) at several distinct time points after induction of gene expression. Arrows indicate the positions of tiny magnetite crystals, while bigger crystals are not labeled. Scale bars: 500 nm.
(TIF)

S9 Fig Effects on MamC-EGFP localization after induction of *mamL* expression. The magnetosome marker protein MamC-EGFP was constitutively co-expressed (in-frame allelic replacement) during induction of *mamL* expression in Δ*mamL mamC*-*egfp* P_*lac*_-*mamL*. The localization gradually developed from a bright punctuate to a predominant linear signal in the first six hours after start of induction. Samples were taken 0, 3, 6 and 22 hours after 2 mM IPTG induction. (A): Quantitative analysis of MamC-EGFP localization 0, 3 and 6 hours after induction. Fluorescent signals in around 200 cells where analyzed for each time point and classified into different localization patterns which are exemplified in representative micrographs of different time points in (B): Single bright spots (asterisk), bright spot and chain (asterisk + arrow), patches (hash) and chains (arrow). Scale bar: 5 μm.
(TIF)

S10 Fig Time-lapse live-cell fluorescence microscopy of Δ*mamB* P_*lac*_-*mamB-egfp*. More detailed representation of Fig 6C. The strain was induced with 2 mM IPTG, transferred to 1% agarose pads containing modified FSM medium and 3 mM IPTG, sealed and incubated at 30°C. Images were acquired every 15 min. The depicted micrographs were acquired in 1 h intervals. Bright field and fluorescence channels are shown. Scale bar: 2 μm.
(TIF)

S1 Video Cryo-electron tomogram of MSR-1 wildtype cell. Tilt images were obtained with direct detector camera, original image pixel size: 0.52 nm.
(M4V)

S2 Video Cryo-electron tomogram of MSR-1 wildtype cell cultivated under non-standard conditions with vigorous shaking at atmospheric oxygen concentrations. Tilt images were obtained with CCD camera, original image pixel size: 0.81 nm. Partially segmented (outer and inner membranes: blue, magnetosome membrane: yellow, iron mineral particles: red).
(M4V)

S3 Video Cryo-electron tomogram of Δ*mamI* cell. Tilt images were obtained with CCD camera, original image pixel size: 0.81 nm. Partially segmented (outer and inner membranes: blue, magnetosome filament: green, magnetosome membrane: yellow, iron mineral particles: red).
(M4V)

S4 Video Cryo-electron tomogram of Δ*mamN* cell. Tilt images were obtained with CCD camera, original image pixel size: 0.81 nm. Partially segmented (outer and inner membranes: blue, magnetosome filament: green, magnetosome membrane: yellow, magnetite crystals: red).
(M4V)

S5 Video Cryo-electron tomogram of Δ*mamL* cell. Tilt images were obtained with direct detector camera, original image pixel size: 0.52 nm. Partially segmented (outer and inner membranes: blue, magnetosome filament: green, magnetosome membrane: yellow, dense magnetosome membrane-like structures: dark yellow, magnetite crystals: red).
(M4V)

S6 Video Cryo-electron tomogram of MSR-1 Δ*mamB* cell. Tilt images were obtained with CCD camera, original image pixel size: 0.71 nm. Partially segmented (outer and inner membranes: blue, magnetosome filament: green, potential dense magnetosome membrane-like structures: dark yellow).
(M4V)

S7 Video Cryo-electron tomogram of Δ*mamQ* cell. Tilt images were obtained with direct detector camera, original image pixel size: 0.52 nm. Partially segmented (outer and inner membranes: blue, magnetosome filament: green, magnetosome membrane: yellow, dense magnetosome membrane-like structures: dark yellow).
(M4V)

S8 Video Cryo-electron tomogram of MSR-1 Δ*mamM* cell. Tilt images were obtained with CCD camera, original image pixel size: 0.71 nm. Partially segmented (outer and inner membranes: blue, magnetosome filament: green, magnetosome membrane: yellow, dense magnetosome membrane-like structures: dark yellow).
(M4V)

S9 Video Time-lapse live-cell fluorescence microscopy with several cells of Δ*mamB* P_*lac*_-*mamB-egfp*. The strain was induced with 2 mM IPTG, transferred to 1% agarose pads containing modified FSM medium and 3 mM IPTG, sealed and incubated at 30°C. Frames were acquired in 15 min intervals. Fluorescence channel is shown.
(AVI)

S10 Video Time-lapse live-cell fluorescent microscopy of Δ*mamB* P_*lac*_-*mamB-egfp* (detail of S9 Video, source of Fig 6 and S9 Fig). Fluorescence channel is shown.
(AVI)

S1 Table Summary of magnetosome membrane phenotypes obtained by analyzing cryo-electron tomography data of different MSR-1 mutant cells. MM: magnetosome membrane, DMM: dense magnetosome membrane-like structure.
(DOCX)

S2 Table Bacterial strains used in this study. (DOCX)

S3 Table Plasmids used in this study. (DOCX)

S4 Table Oligonucleotides used in this study. (DOCX)
