# Supplementary material for: Genetic and Ultrastructural Analysis Reveals the Key Players and Initial Steps of Bacterial Magnetosome Membrane Biogenesis
Source: PLoS Genet. 2016 Jun 10;12(6):e1006101. doi: 10.1371/journal.pgen.1006101 (PMC4902198; doi:10.1371/journal.pgen.1006101)
Supplement: S1 Text — (DOCX) [file pgen.1006101.s001.docx]

# S1 Text. Amino acid substitutions within MamL

MamL contains nine basic and potentially positively charged (including histidine) amino acid residues close to or at its very C-terminus. The C-terminal accumulation of basic residues is a conserved feature in MamL and MamL-like homologs from other MTB (S5 Fig). In order to analyze if these residues play a role for protein localization or magnetite biomineralization, we expressed a MamL-EGFP fusion in Δ*mamL* and also substituted the positively charged residues in MamL-EGFP to six different combinations to structurally similar but neutral amino acids [(a) K77Q R78Q; (b) K72Q; (c) K63Q K66Q K68Q; (d) H67Y; (e) R64Q R65Q; (f) all nine point mutations combined (MamL_all neutral_)]. Complementation with wild type MamL-EGFP rescued 62% and 66% of wild type magnetosome number and diameter respectively, while expression of MamL_all neutral_-EGFP did not enhance particle formation at 30°C and thus phenocopied the ∆*mamL* mutant. All other point mutants caused a slight or intermediate decrease in magnetite crystal number and size as compared to the *mamL*-e*gfp* control (Fig 4A). MamL-EGFP localized in (short) linear fluorescent signals in 50% of the analyzed cells, indicating an at least partial magnetosome localization of the fusion protein (Fig 4A). In the single amino acid substituted MamL-GFP strains, also 25% to 42% of the cells showed a linear localization pattern (Fig 4A). While not restoring biomineralization, MamL_all neutral_-GFP surprisingly localized in a chain-like pattern in 48% of the cells as well (Fig 4A), suggesting that the positively charged C-terminal residues are not involved in MM-tubulation or -interaction but rather participate in a function related to magnetite maturation.
