# Supplementary material for: Genetic and Ultrastructural Analysis Reveals the Key Players and Initial Steps of Bacterial Magnetosome Membrane Biogenesis
Source: PLoS Genet. 2016 Jun 10;12(6):e1006101. doi: 10.1371/journal.pgen.1006101 (PMC4902198; doi:10.1371/journal.pgen.1006101)
Supplement: S2 Text — (DOCX) [file pgen.1006101.s002.docx]

# S2 Text. Amino acid substitutions within MamQ

MamQ shows homology to the widespread, but so far non-characterized LemA protein family of which another homolog (63% similarity, LemA_MSR_ [MGR_0326 or MGMSRv2_3349]) is encoded outside the MAI of MSR-1. We deleted *lemA_MSR-1_* and could not find an aberrant magnetosome phenotype, indicating that the gene is irrelevant for magnetosome formation. LemA from *Thermotoga maritima* is the only homolog of MamQ (46% similarity) with an experimentally determined crystal structure. The soluble part of the protein (PDB ID 2ETD) shows a 4-helix bundle which is attached to a predicted transmembrane helix. LemA_MSR_/LemA*_T.maritima_* and MamQ_MSR_ differ mainly in their N-terminal regions and a MamQ specific stretch within the predicted helix bundles (S6 Fig). Based on the crystal structure of LemA*_T.maritima_*, we obtain a structural MamQ_MSR_ model and identified several conserved residues [E111; E179, Y181, Y241, F242] shared by all analyzed MamQ and LemA homologs and which are putatively exposed on the surface in loop regions of the helix bundle or on a flexible C-terminal region (S6 Fig and S7 Fig). To analyze their role for the *in vivo* function of the protein, we substituted the mentioned residues by allelic replacement in 5 different combinations to neutral amino acids [1: Y241A F242A; 2: E179A; 3: Y181A; 4: E111A; 5: E179A Y181A E111A] in *mamQ*::*egfp-mamQ*. Expression of the mutated *egfp-mamQ* versions was indicated by a weak fluorescence signal in patches along the cytoplasmic membrane (S7 Fig). However, none of the mutants showed a restoration of regular magnetosome formation as analyzed by a C_mag_ of 0 and no detectable magnetite crystals by conventional TEM, in contrast to an almost wild type-like C_mag_ (1.75) of the non-mutated e*gfp-mamQ* complementation strain. These results indicate the importance of these putatively surface-exposed conserved residues for the function of MamQ.
