# Supplementary material for: Genetic and Ultrastructural Analysis Reveals the Key Players and Initial Steps of Bacterial Magnetosome Membrane Biogenesis
Source: PLoS Genet. 2016 Jun 10;12(6):e1006101. doi: 10.1371/journal.pgen.1006101 (PMC4902198; doi:10.1371/journal.pgen.1006101)
Supplement: S3 Text — (DOCX) [file pgen.1006101.s003.docx]

# S3 Text. Supplemental experimental procedures

**Plasmid construction***Plasmids for fluorescent magnetosome protein fusions*

By integrating oligonucleotides oOR164 and 165, coding for a stable 25 aa alpha-helical linker region [LA(EAAAK)_4_AAA] (HL) [1], upstream or downstream of MSR-1-optimized *egfp* [2] into the pBBR1MCS2 derivate pOR071, we developed a platform for N- or C- terminal EGFP fusions under control of the strong P*_mamDC45_* promotor. The genes *mamI* (C-terminal fusion, primers: oOR155 and 170), *mamL* (C-terminal fusion; neutrally point mutated to remove internal *NdeI* restriction site, primers: oOR157 and 171) and *mamQ* (N-terminal fusion; neutrally point mutated to remove internal *NdeI* restriction site, primers: oOR180 and 181) were amplified from genomic MSR-1 DNA and cloned into this platform to create plasmids pOR075, pOR077 and pOR086. For stable genomic integrations, we additionally cloned the P*_mamDC_* *egfp-*HL*-mamQ* and P*_mamDC_* *mamL-*HL*-egfp* expression cassettes in between the Tn5 ends of pBAM-1 by restriction digestion to create pOR150 and pOR151. By subcloning of P*_mamDC_* *mamL-*HL*-egfp* and PCR-based site directed PCR mutagenesis before re-integration into pOR151, we created pBAM-based plasmids pOR163-167 for point mutation analysis. For construction of pOR168, harboring P*_mamDC_* *mamL*_[all neutral]_*-*HL*-egfp*, the point mutated *mamL* gene was fully synthesized (ATG:biosynthetics GmbH, Germany) and cloned into pOR151.

The plasmids for genomic in-frame integration of e*gfp-mamQ* were created by amplifying and fusing DNA fragments upstream of *mamQ*, and *egfp*-HL-*mamQ* (primers: oOR264-267) and by cloning them into pORFM galK [3] to create pYF001. An analogous procedure was used to create pORFM B-GFP. Point mutations in *egfp*-HL-*mamQ* in plasmids pYF003-007 were introduced by subcloning and site directed PCR mutagenesis (primers: oOR296-305). By overlap extension PCR cloning [4] from pYF001, *egfp* in *egfp*-HL-*mamQ* was replaced by *mCherry* to create pYF002 (primers: oOR278 and 279).

*Integrative plasmids for expression of artificial mam operons*

Plasmids for the expression of *mamLQRB* (pOR140) and *mamLMQRB* (pOR155) were constructed by amplification of *mamL* from pOR077 using primer pair oOR171/359 and amplification of *mamQRB* from genomic MSR-1 DNA using primer pair oOR360/361, followed by fusion and cloning of both fragments into a pBAM-1 derived vector. The P*_mamDC_* promotor in the plasmid was exchanged by P*_mamAB_*_,_ (oligonucleotides oOR357/358) creating pOR140. *mamLM* was amplified using oOR171/375 and cloned into pOR140 to replace *mamL*, creating pOR155. For generation of a minimal magnetosome gene cluster, two artificially arranged operons were designed and synthesized (ATG:biosynthetics GmbH). The two building blocks comprised the genes (i) *mamL*, *mamB*, *mamQ* and (ii) *mamI*, *mamE*, *mamM*, *mamO*. Both operons were put under transcriptional control of the *mamAB* promoter (P*_mamAB_*). Artificial Shine Dalgarno sequences with free accessibility on the messenger RNA were designed according to different computational models [5–7]. The coding sequences were not further optimized except for removal of disruptive endonuclease restriction sites. To allow the future complete de-assembly, recovery as well as recycling of all genes, individual coding sequences were each separated by an ABM (AscI-BssHII-MauBI) restriction site. The artificial expression cassettes were cloned in between the Tn5 ends of pBAM-1 to create pBAM_minMAI.

*Integrative plasmids for induction of magnetosome gene expression*

The following system was designed for the induction of single magnetosome gene expression in the respective gene-deletion background strain. To ensure reliably inducible, but tightly controlled expression, we used the *lac* promoter - *lacI* repressor system, which was shown to be functional in MSR-1 [8,9] and combined it with a chromosomally integrative vector. To avoid potential undesired expression upon random genomic insertion, we utilized the site-specific Tn7 transposon system [10], which integrated specifically and reliably into a genomic region around the *glmS* gene in MSR-1. The MSR-adapted Tn7 transposable pre-plasmid for magnetosome protein induction (pT18-Tn7T-Km) was created as following: A minimized pT18mob2 vector backbone was amplified using primers pT18mob2+Tet_for_NotI and pT18mob2_rev_NcoI, digested and ligated with a mini-Tn7 fragment that was amplified from plasmid pUC18R6K-mini-Tn7T-Km using primers Tn7Km_for and Tn7Km_rev. The MSR-adapted transposase-encoding helper plasmid pT18mob2PmamDC-TnsAD was obtained by digestion of the transposase (TnsA) containing fragment from pTns2 and cloning into pT18mob2 vector. A P*_mamDC_*-TnsA fragment was created by two-step fusion-PCR using primer pairs TnsA for/rev and PmamDCrev/PmamDC96 and subsequently cloned into the vector to replace P*_lac_*-TnsA. Plasmids for induction of *mamL*, *mamB* and *mamB-egfp* expression were constructed by first amplifying the genes, using primer pairs oOR171/282, oOR 386/387 for *mamL* and *mamB*, respectively and cloning them into pFM211 [8]. *mamB* and HL-*egfp* were amplified using primer pairs oOR420/421 and oOR422/423 and fused in a two-step PCR. The created plasmids were named pOR117, pOR158 and pOR169. The constructs, containing P*_lac_*-*mamL*/*mamB/mamB-GFP* and *lacI* were cloned in between the Tn7 ends Tn7L and Tn7R in pT18-Tn7T-Km to create pOR118, pOR160 and pOR171, respectively. Plasmid transfer into MSR-1 was mediated by triparental mating.

**Cell fractionation, SDS-gel electrophoresis and immunological detection**

Cellular fractionation of MSR-1 and magnetosome purification were performed essentially as previously described using a magnetized separation column [11,12]. SDS-PAGE was performed as previously described [12]. Protein concentrations were determined using BCA-Protein Micro assay (Pierce) and normalized fraction samples (approx. 2 µg protein) were supplemented with electrophoresis buffer and heated to 95°C for 5 min. These samples or whole cell samples from the induction experiment, respectively, were subjected to 12% polyacrylamide gels. For western blot analysis, proteins were electro-blotted on PVDF membranes. Immunological protein detection was performed as described [12]. In this study, Anti-MamB [12] and Anti-RFP [5F8] (ChromoTek GmbH, Martinsried, Germany) were used as primary antibodies.

**Periplasmic diffusion experiments**

In search for a suitable export signal for the twin-arginine translocon, we examined the genomes of *Escherichia coli* and MSR-1 for suitable candidates. We found TorA from E. coli and NapA and the Ni,Fe-hydrogenase I small subunit MGR_0500 from MSR-1 as suitable candidates. Extend of the export signals (RR) was analyzed by the TatP prediction server (<http://www.cbs.dtu.dk/services/TatP/>). We amplified the respective DNA sequences from genomic DNA and fused them with *egfp*. Expressed from pBBR plasmids and the P_lac_/P_mamDC_ promoter, all RR-GFP versions were partially transported into the periplasmic space of *E. coli* and MSR-1. Highest fluorescence in the periplasmic space of MSR-1 was obtained with the RR-signal from MGR_0500, which was utilized for final fluorescence microscopy and western-blot experiments. The utilized plasmid pOR082 was created by cloning RR (MGR0500)-*egfp* (Fusion PCR using primer oOR113/134 and oOR130/146) into pOR071. For experiments with 5(6) Carboxyfluorescein (FAM), MSR-1 wild type cells were incubated overnight with FSM medium containing 1 mM (5)6 FAM. The cells showed normal growth. Cell were harvested by centrifugation and either 3x washed in 1 volume of PBS or previously chemically fixed by addition of 0.075% formaldehyde and 5 mg/mL BSA for 15 min before washing in order to entrap the molecule within the cells (control). Cells were prepared on agarose slices for fluorescence microscopy.

**Bioinformatic tools**

The protein structure of MamQ was modelled with SWISS-MODEL [13] using amino acids 70-246 on Template 2ETD (PDB ID) and displayed with Jmol 3.7 (<http://www.jmol.org/>). Proteins were analyzed using SMART [14] and the NCBI database (<http://www.ncbi.nlm.nih.gov/>). Sequence alignments were performed with clustalΩ (<http://www.ebi.ac.uk/Tools/msa/clustalo/>).
