# Supplementary material for: Genetic and Ultrastructural Analysis Reveals the Key Players and Initial Steps of Bacterial Magnetosome Membrane Biogenesis
Source: PLoS Genet. 2016 Jun 10;12(6):e1006101. doi: 10.1371/journal.pgen.1006101 (PMC4902198; doi:10.1371/journal.pgen.1006101)
Supplement: S4 Text — (DOCX) [file pgen.1006101.s004.docx]

# S4 Text References cited in supporting information.

1. Arai R, Ueda H, Kitayama A, Kamiya N, Nagamune T. Design of the linkers which effectively separate domains of a bifunctional fusion protein. Protein Eng. 2001;14: 529–32. Available: http://www.ncbi.nlm.nih.gov/pubmed/11579220

2. Borg S, Hofmann J, Pollithy A, Lang C, Schüler D. New Vectors for Chromosomal Integration Enable High-Level Constitutive or Inducible Magnetosome Expression of Fusion Proteins in Magnetospirillum gryphiswaldense. Appl Environ Microbiol. 2014;80: 2609–16. doi:10.1128/AEM.00192-14

3. Raschdorf O, Plitzko JM, Schüler D, Müller FD. A tailored galK counterselection system for efficient markerless gene deletion and chromosomal tagging in Magnetospirillum gryphiswaldense. Appl Environ Microbiol. 2014;80: 4323–4330. doi:10.1128/AEM.00588-14

4. Bryksin A V, Matsumura I. Overlap extension PCR cloning: a simple and reliable way to create recombinant plasmids. Biotechniques. 2010;48: 463–5. doi:10.2144/000113418

5. Salis HM, Mirsky EA, Voigt CA. Automated design of synthetic ribosome binding sites to control protein expression. Nat Biotechnol. 2009;27: 946–950. Available: http://dx.doi.org/10.1038/nbt.1568

6. Andronescu M, Condon A, Hoos HH, Mathews DH, Murphy KP. Computational approaches for RNA energy parameter estimation. RNA. 2010;16: 2304–2318. doi:10.1261/rna.1950510

7. Mathews DH. Using an RNA secondary structure partition function to determine confidence in base pairs predicted by free energy minimization. RNA. United States; 2004;10: 1178–1190. doi:10.1261/rna.7650904

8. Müller FD, Raschdorf O, Nudelman H, Messerer M, Katzmann E, Plitzko JM, et al. The FtsZ-Like Protein FtsZm of Magnetospirillum gryphiswaldense Likely Interacts with Its Generic Homolog and Is Required for Biomineralization under Nitrate Deprivation. J Bacteriol. 2014;196: 650–9. doi:10.1128/JB.00804-13

9. Lang C, Pollithy A, Schüler D. Identification of promoters for efficient gene expression in Magnetospirillum gryphiswaldense. Appl Environ Microbiol. 2009;75: 4206–10. doi:10.1128/AEM.02906-08

10. Lambertsen L, Sternberg C, Molin S. Mini-Tn7 transposons for site-specific tagging of bacteria with fluorescent proteins. Environ Microbiol. 2004;6: 726–732. doi:10.1111/j.1462-2920.2004.00605.x

11. Grünberg K, Müller E-C, Otto A, Reszka R, Linder D, Kube M, et al. Biochemical and Proteomic Analysis of the Magnetosome Membrane in Magnetospirillum gryphiswaldense. Appl Environ Microbiol. 2004;70: 1040–1050. doi:10.1128/AEM.70.2.1040-1050.2004

12. Uebe R, Junge K, Henn V, Poxleitner G, Katzmann E, Plitzko JM, et al. The cation diffusion facilitator proteins MamB and MamM of Magnetospirillum gryphiswaldense have distinct and complex functions, and are involved in magnetite biomineralization and magnetosome membrane assembly. Mol Microbiol. 2011;84: 818–835. doi:10.1111/j.1365-2958.2011.07863.x

13. Biasini M, Bienert S, Waterhouse A, Arnold K, Studer G, Schmidt T, et al. SWISS-MODEL: Modelling protein tertiary and quaternary structure using evolutionary information. Nucleic Acids Res. 2014;42: 252–258. doi:10.1093/nar/gku340

14. Letunic I, Copley RR, Schmidt S, Ciccarelli FD, Doerks T, Schultz J, et al. SMART 4.0: towards genomic data integration. Nucleic Acids Res. 2004;32: D142–4. doi:10.1093/nar/gkh088

15. Schultheiss D, Schüler D. Development of a genetic system for Magnetospirillum gryphiswaldense. Arch Microbiol. 2003;179: 89–94. doi:10.1007/s00203-002-0498-z

16. Ullrich S, Schüler D. Cre-lox-based method for generation of large deletions within the genomic magnetosome island of Magnetospirillum gryphiswaldense. Appl Environ Microbiol. 2010;76: 2439–44. doi:10.1128/AEM.02805-09

17. Schübbe S, Kube M, Scheffel A, Wawer C, Heyen U, Meyerdierks A, et al. Characterization of a Spontaneous Nonmagnetic Mutant of Magnetospirillum gryphiswaldense Reveals a Large Deletion Comprising a Putative Magnetosome Island. J Bacteriol. 2003;185: 5779–5790. doi:10.1128/JB.185.19.5779-5790.2003

18. Lohße A, Borg S, Raschdorf O, Kolinko I, Tompa É, Pósfai M, et al. Genetic dissection of the mamAB and mms6 operons reveals a gene set essential for magnetosome biogenesis in magnetospirillum gryphiswaldense. J Bacteriol. 2014;196: 2658–2669. doi:10.1128/JB.01716-14

19. Simon R, Priefer U, Pühler A. A Broad Host Range Mobilization System for In Vivo Genetic Engineering: Transposon Mutagenesis in Gram Negative Bacteria. Nat Biotechnol. 1983;1: 784–791. doi:10.1038/nbt1183-784

20. Martínez-García E, Calles B, Arévalo-Rodríguez M, de Lorenzo V. pBAM1: an all-synthetic genetic tool for analysis and construction of complex bacterial phenotypes. BMC Microbiol. BioMed Central Ltd; 2011;11: 38. doi:10.1186/1471-2180-11-38

21. Kovach ME, Elzer PH, Hill DS, Robertson GT, Farris MA, Roop RM, et al. Four new derivatives of the broad-host-range cloning vector pBBR1MCS, carrying different antibiotic-resistance cassettes. Gene. 1995;166: 175–176. doi:10.1016/0378-1119(95)00584-1

22. Kirchner O, Tauch A. Tools for genetic engineering in the amino acid-producing bacterium Corynebacterium glutamicum. J Biotechnol. Netherlands; 2003;104: 287–299.

23. Choi K-H, Gaynor JB, White KG, Lopez C, Bosio CM, Karkhoff-Schweizer RR, et al. A Tn7-based broad-range bacterial cloning and expression system. Nat Methods. 2005;2: 443–448. doi:10.1038/nmeth765

24. Raschdorf O, Müller FD, Pósfai M, Plitzko JM, Schüler D. The magnetosome proteins MamX, MamZ, and MamH are involved in redox control of magnetite biomineralization in Magnetospirillum gryphiswaldense. Mol Microbiol. 2013;89: 872–886. doi:10.1111/mmi.12317
