# Supplementary figures and images for: Genetic and Ultrastructural Analysis Reveals the Key Players and Initial Steps of Bacterial Magnetosome Membrane Biogenesis
Source: PLoS Genet. 2016 Jun 10;12(6):e1006101. doi: 10.1371/journal.pgen.1006101 (PMC4902198; doi:10.1371/journal.pgen.1006101)

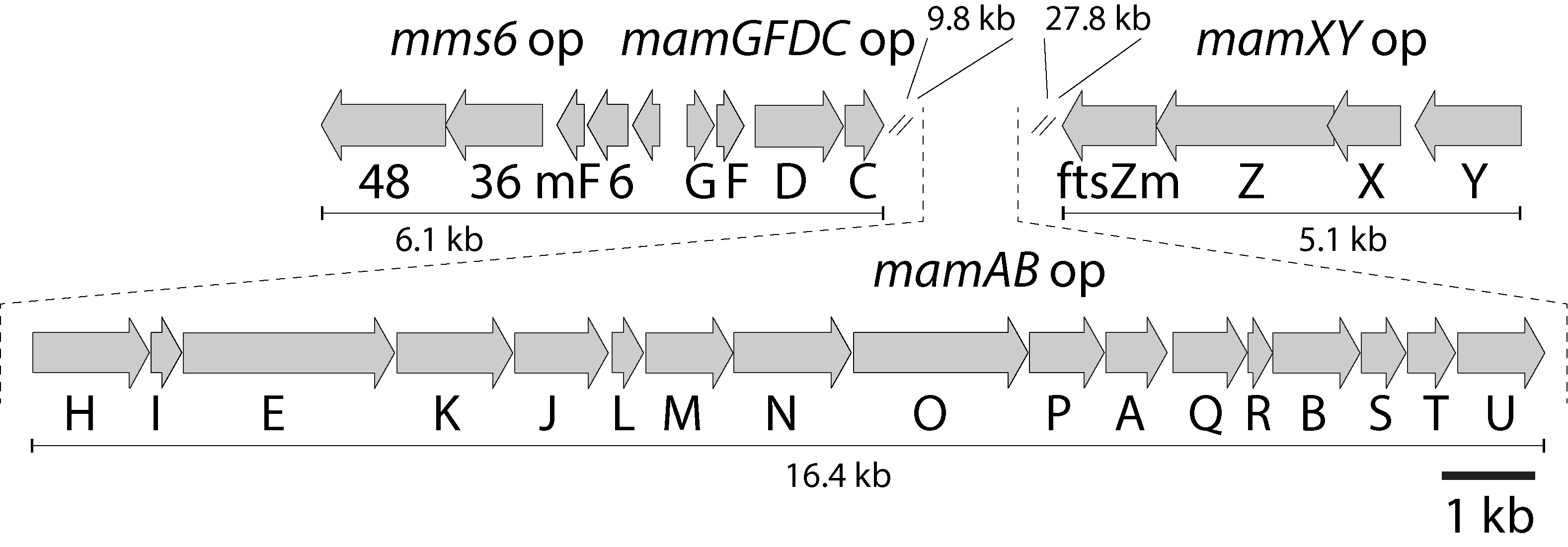

Supplement: S1 Fig — The mamAB operon comprises 17 genes (from mamH to mamU), the mamGFDC operon (from mamG to mamC), the mms6 operon (mms6, mmsF, mms36 and mms48) and the mamXY operon (mamY, mamX, mamZ and ftsZm) each comprise 4 relevant genes. The extend of the regions separating the individual operons is indicated in kilobase pairs (kb). (TIF) [file pgen.1006101.s005.tif]

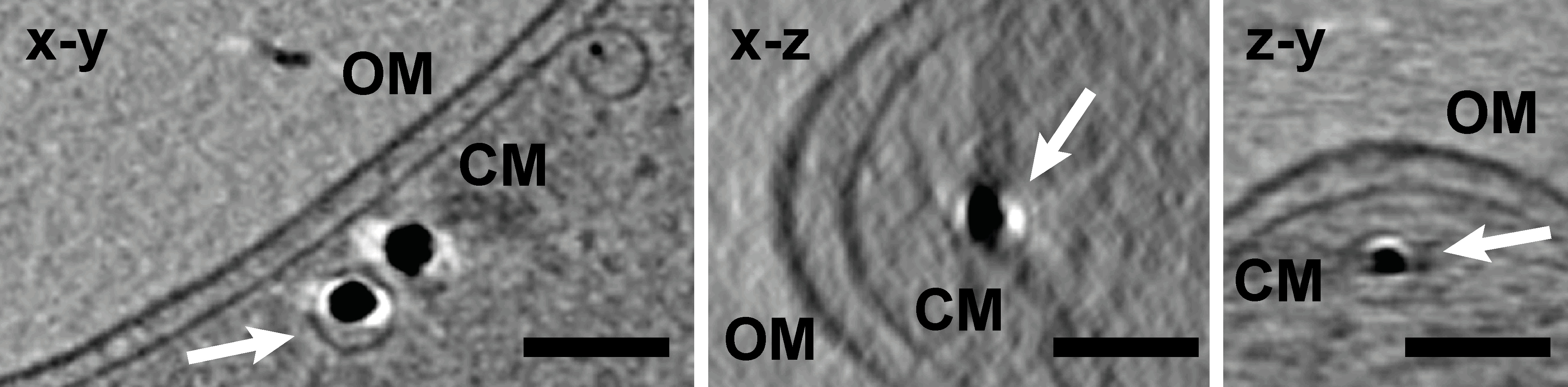

Supplement: S2 Fig — Image slices (x-y; x-z; z-y) from cryo-electron tomogram of MSR-1 wildtype. The same magnetite-containing magnetosome membrane vesicle is indicated in all three image slices by white arrows. The vesicle resides within some distance and is clearly disconnected from the cytoplasmic membrane (CM). Outer membrane (OM) is indicated. Scale bar: 100 nm. (TIF) [file pgen.1006101.s006.tif]

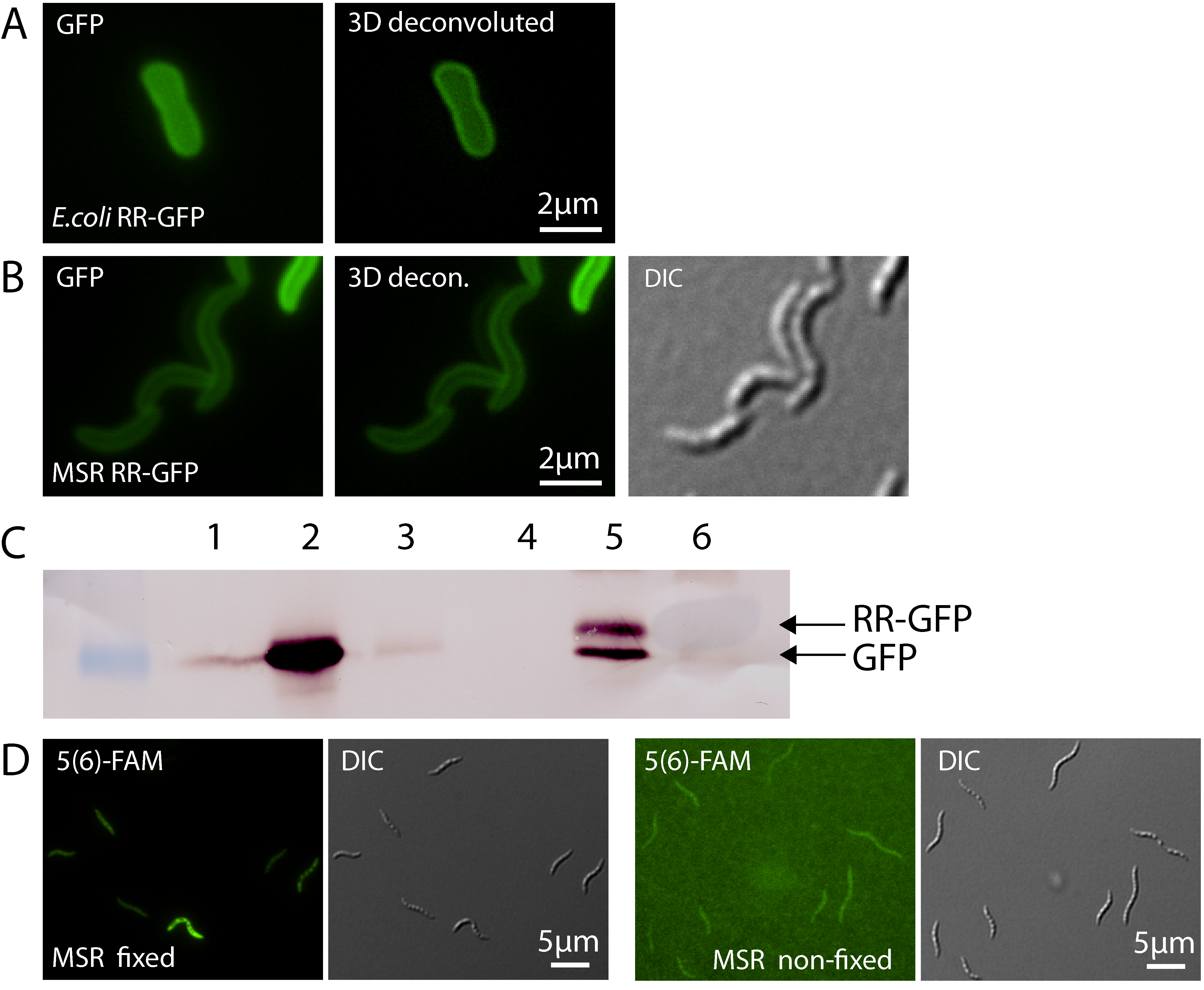

Supplement: S3 Fig — The Twin Arginine Translocation (TAT) signal peptide (RR) of MSR-1 protein MGR0500 was fused to EGFP and the construct expressed in E. coli strain BW29427 and MSR-1. Fluorescent micrographs show that the modified EGFP was efficiently translocated into the periplasmic space of (A) E. coli and (B) MSR-1. No linear signal was detected within MSR-1, indicating lack of diffusion and entrapment of the protein in the MM. Left: green channel, middle: 3D-deconvoluted representation, right (in B): DIC channel. (C): Western blot with purified and protein concentration normalized fractions from MSR-1 expressing EGFP (lanes 1–3) or RR-EGFP (lanes 4–6). MM protein fraction (lane 1 and 4), total soluble protein fraction (lane 2 and 5) and total cellular membrane protein fraction (lane 3 and 6). Immunodetection was performed with GFP Antibody. Arrows indicate a putative signal for GFP and RR-GFP. The RR-signal cleavage after translocation of the protein to the periplasm can be observed in the blot. (D): Assay to determine 5(6) Carboxyfluorescein (FAM)-diffusion into MM. MSR-1 was cultivated over-night in FSM medium supplemented with 1 mM 5(6) FAM. Cells were either 3x washed in 1 volume of PBS or previously chemically fixed by addition of 0.075% formaldehyde and 5 mg/mL BSA for 15 min before washing. Left micrographs shows fixed cells that are fluorescent, indicating enclosure of 5(6) FAM, right micrograph shows unfixed cells that are non-fluorescent. (TIF) [file pgen.1006101.s007.tif]

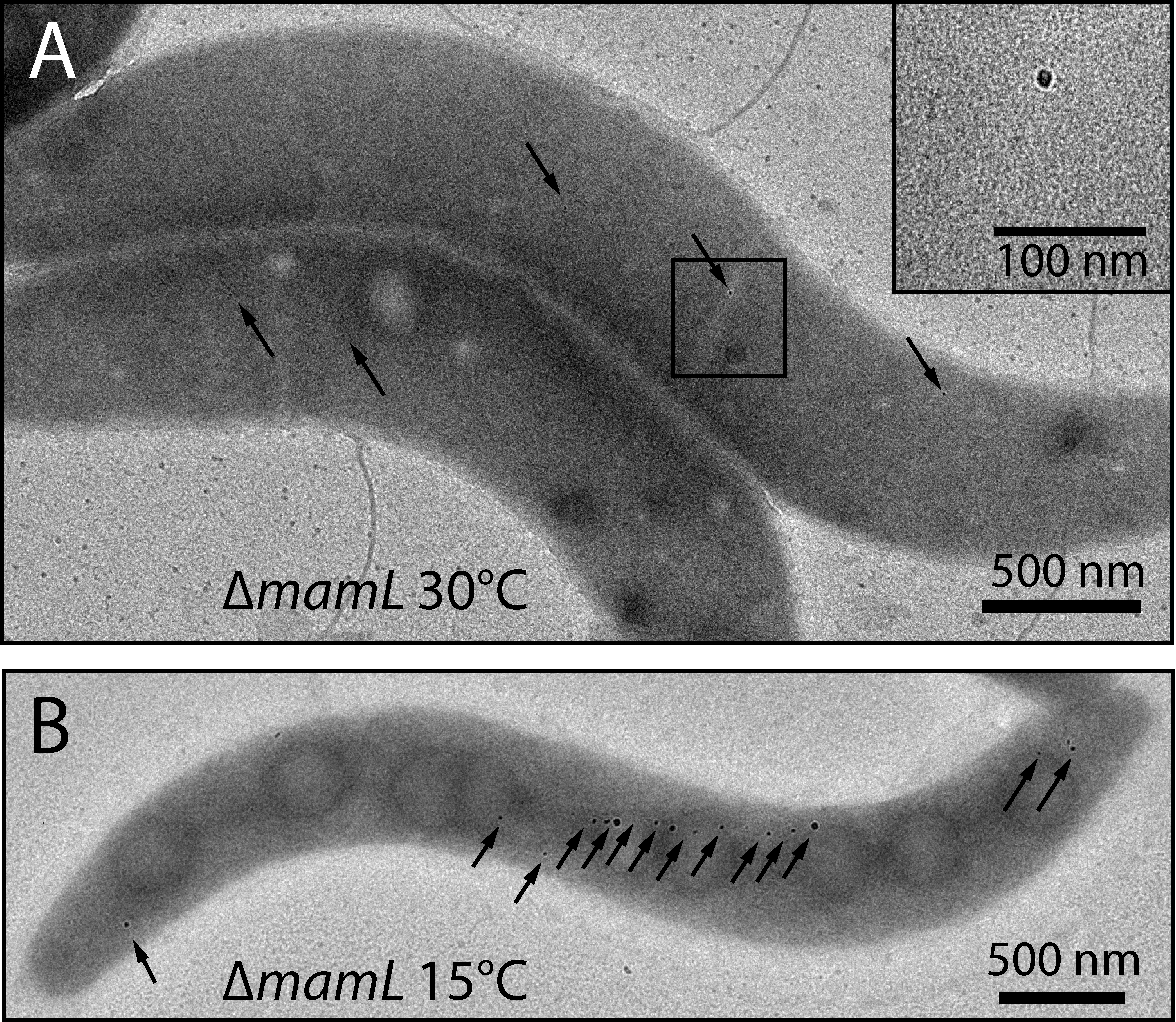

Supplement: S4 Fig — (A): Micrograph of ΔmamL cell, cultivated at 30°C (under standard conditions). Inlet shows indicated area in higher magnification. Arrows indicate position of (putative) tiny magnetite particles (B): Micrograph of ΔmamL cell, cultivation at 15°C. Arrows indicate position of magnetite particles. (TIF) [file pgen.1006101.s008.tif]

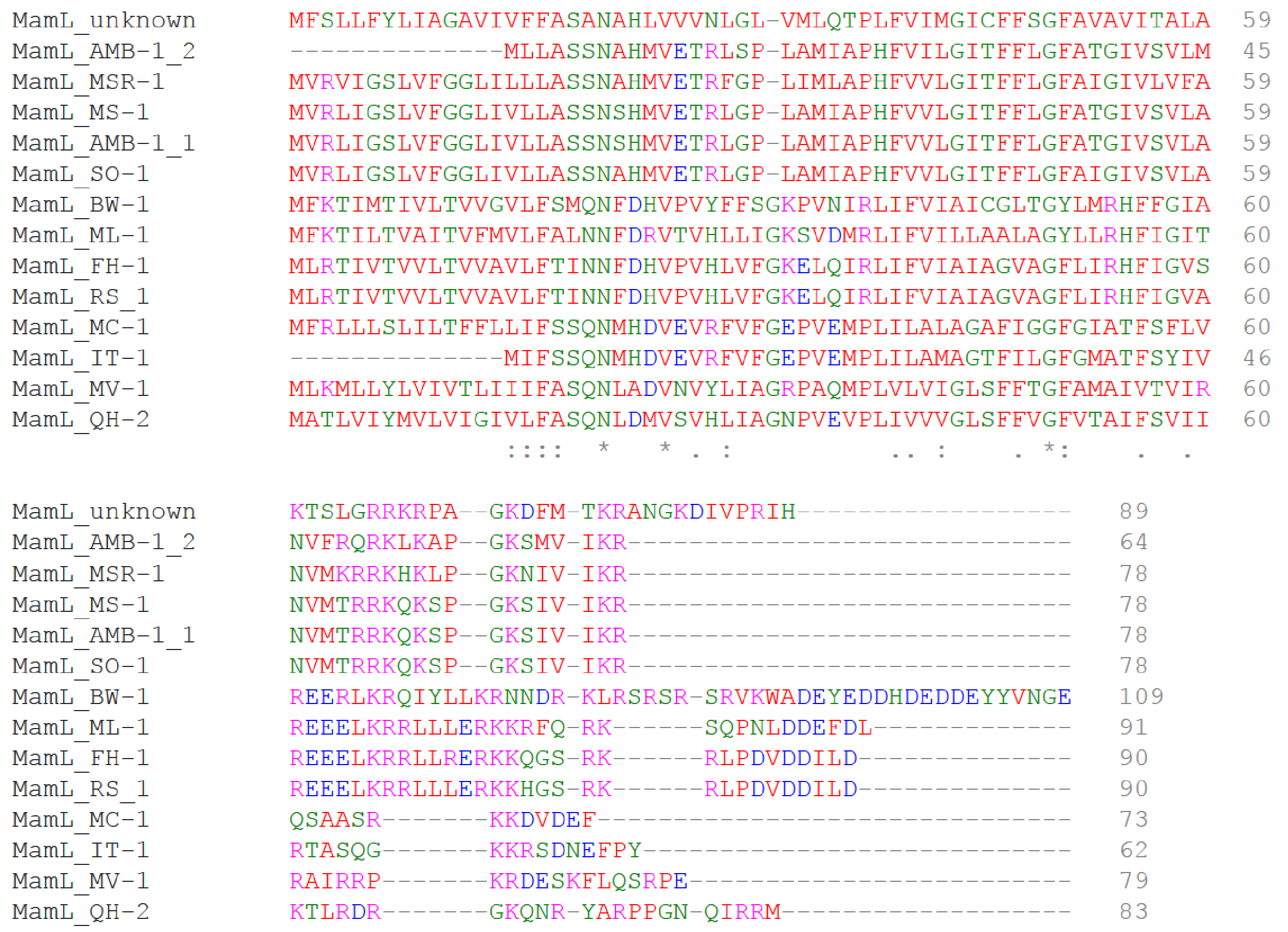

Supplement: S5 Fig — Basic amino acids are indicated in purple and are enriched in C-terminal regions. (TIF) [file pgen.1006101.s009.tif]

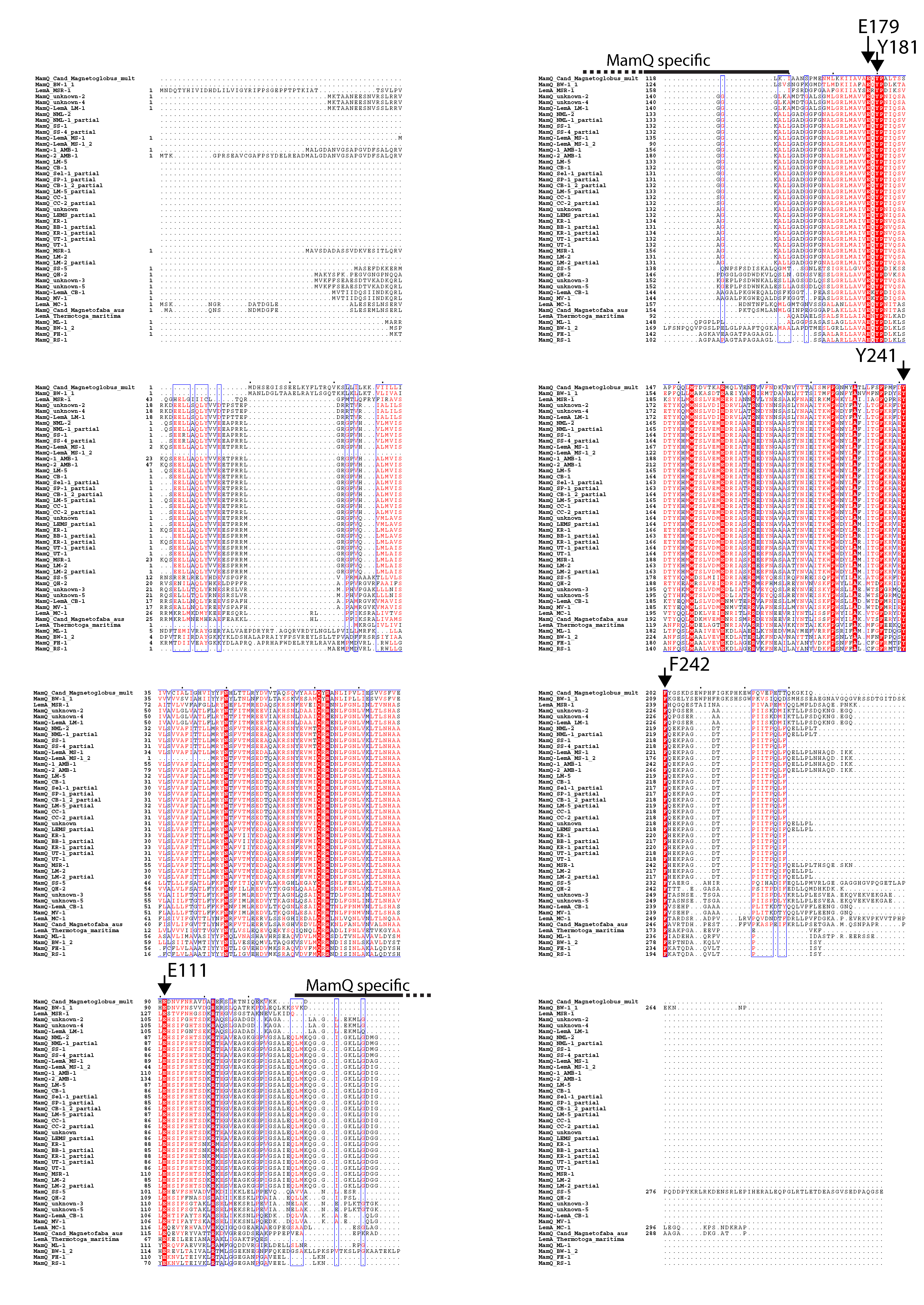

Supplement: S6 Fig — The MTB-specific stretch and the analyzed point mutated residues from this study are indicated. (TIF) [file pgen.1006101.s010.tif]

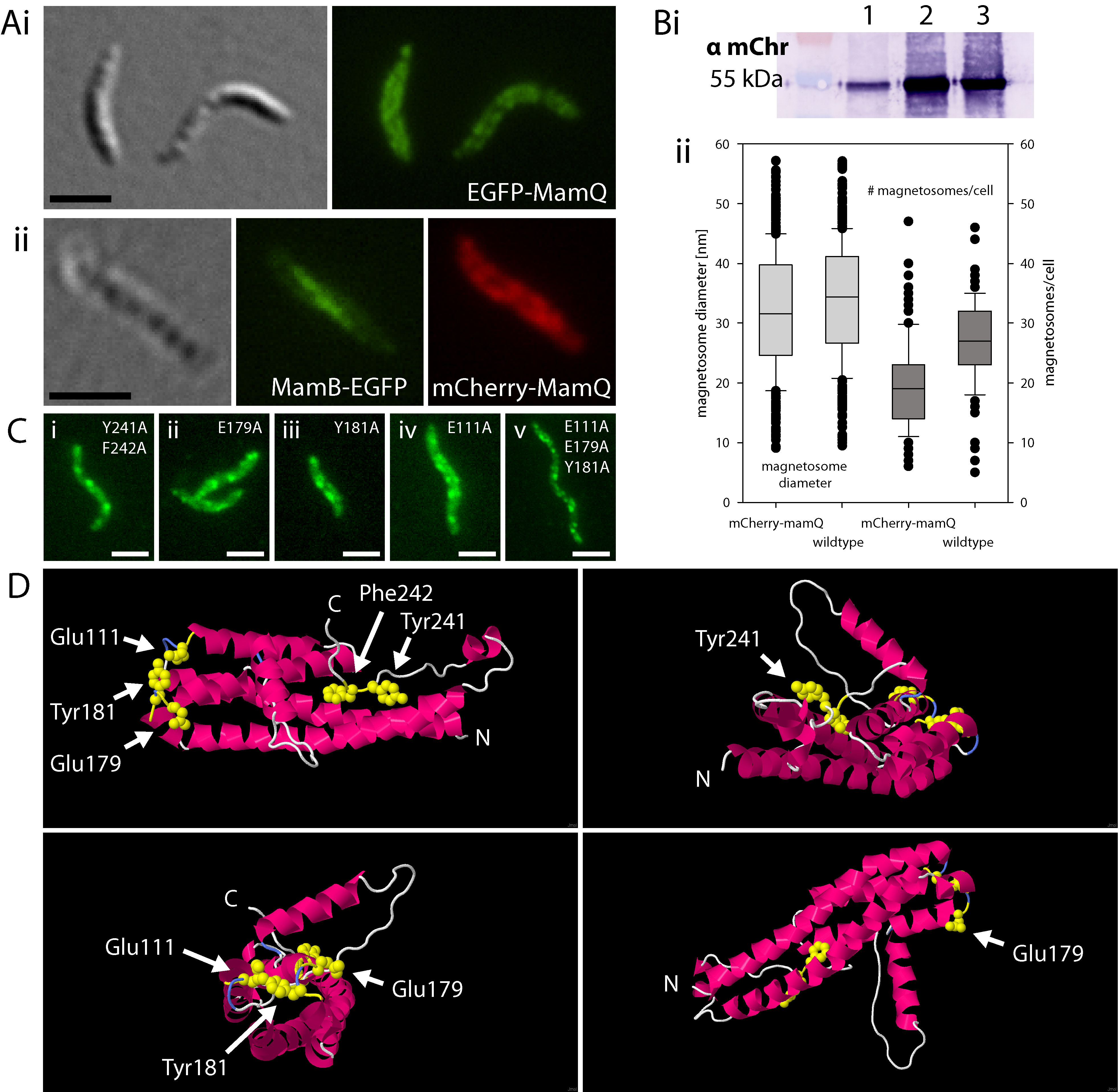

Supplement: S7 Fig — (A): Representative fluorescence micrographs of MSR-1 cells (Ai): overexpressing PmamDC45-mamQ-egfp and (Aii): expressing chromosomal in-frame allelic replacements of mamB::mamB-GFP and mamQ::mCherry-mamQ. From left to right: DIC channel, green fluorescent channel, red fluorescent channel. Scale bars: 2 μm (Bi): Western blot with separated and concentration-normalized fractions obtained from mamQ::mCherry-mamQ cell lysate. Total soluble protein fraction (lane 1), total non-magnetic membrane protein fraction (lane 2) and magnetosome membrane protein fraction (lane 3). Primary immuno-detection was performed with MCherry antibody. (Bii): Quantitative analysis of magnetosome diameter (left) and magnetosome number (right) of mamQ::mCherry-mamQ and wild type. Box plots are indicating 10th and 90th percentiles (whiskers), 25th and 75th percentiles (box), median and outliers. Over 500 magnetosomes and 100 cells where analyzed, each. (C): Fluorescence micrographs of non-magnetic MSR-1 cells expressing in-frame chromosomal replacements of (i) mamQ::egfp-mamQY241A F242A,(ii) mamQ::egfp-mamQE179A, (iii) mamQ::egfp-mamQY181A, (iv) mamQ::egfp-mamQE111A and (v) mamQ::egfp-mamQE111A E179A Y181A. Scale bars: 2 μm. (D): Different views on the model of MamQMSR-1 tertiary structure. The protein structure of the soluble part of MamQ (using amino acids 70–246) was modelled with SWISS-MODEL and the experimentally determined 2.28 Å resolved crystal structure of LemAT.maritima [PDB ID 2ETD] as template (GMQE = 0.30, QMEAN = -6.80). Only the backbone of the structure is visualized. Putative alpha-helical regions are depicted in purple, the side chains of the mutated amino acids in this study are depicted in yellow, represented in stick and ball model and indicated by an arrow if visible in the view. They are either localized in predicted loop or flexible regions. If visible, also the N and C-termini of the modeled protein structure are indicated. The predicted trans-membrane domain of MamQ c [file pgen.1006101.s011.tif]

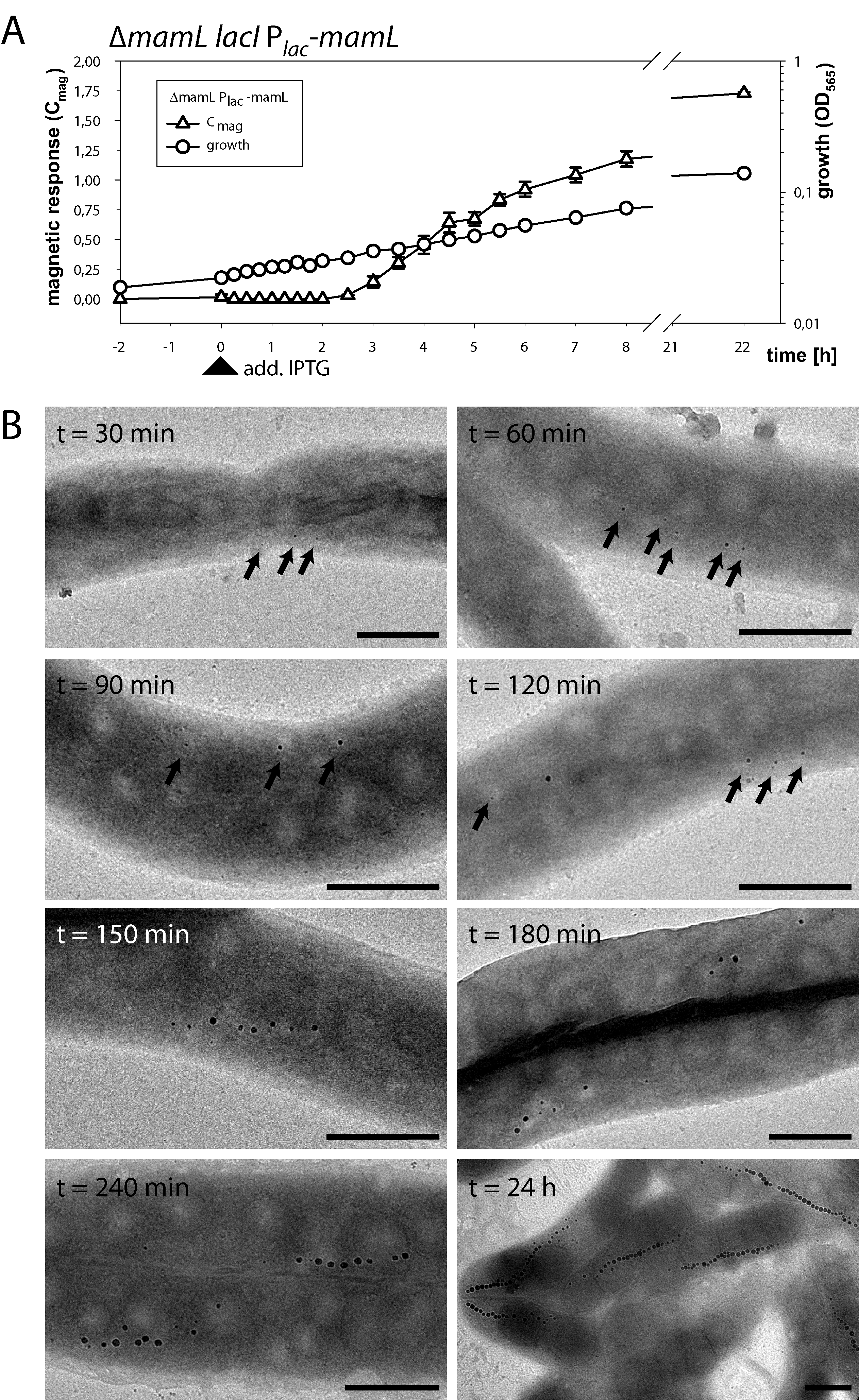

Supplement: S8 Fig — Induction of mamL expression in ΔmamL Plac-mamL. In the absence of the inducer IPTG, the strain failed to exhibit a magnetic response (Cmag = 0) when cultivated at 30°C. Upon addition of IPTG, a gradual restoration of Cmag and magnetosome size and number was detected and wild type-like levels were reached after over-night incubation. (A): Progression of growth (OD565, circles) and magnetic response (Cmag, triangles) over time after induction of mamL expression with 2mM IPTG in ΔmamL Plac-mamL. IPTG was added at time point 0 (black triangle). (B) TEM micrographs showing magnetite crystal morphology in cell from experiment (A) at several distinct time points after induction of gene expression. Arrows indicate the positions of tiny magnetite crystals, while bigger crystals are not labeled. Scale bars: 500 nm. (TIF) [file pgen.1006101.s012.tif]

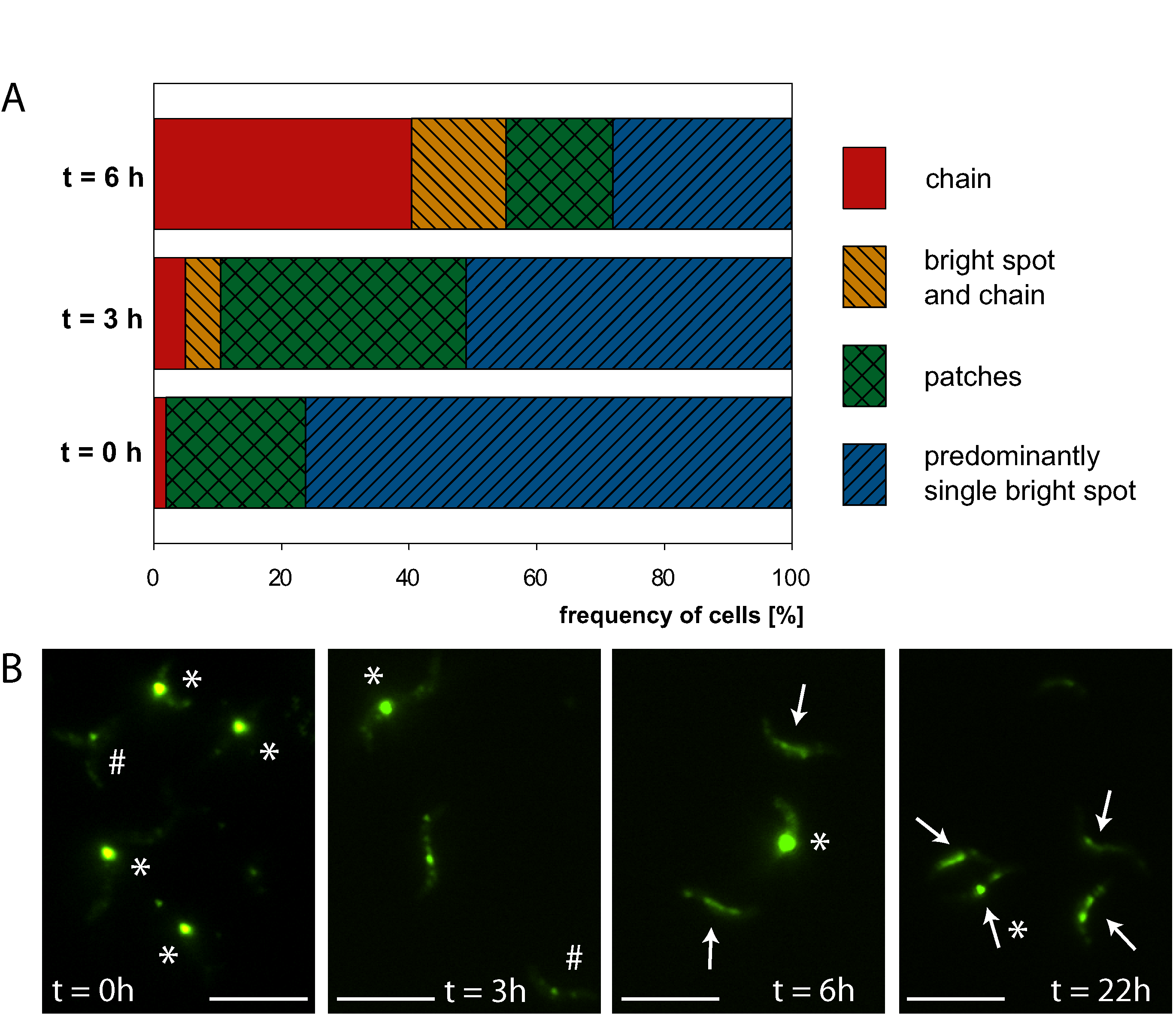

Supplement: S9 Fig — The magnetosome marker protein MamC-EGFP was constitutively co-expressed (in-frame allelic replacement) during induction of mamL expression in ΔmamL mamC-egfp Plac-mamL. The localization gradually developed from a bright punctuate to a predominant linear signal in the first six hours after start of induction. Samples were taken 0, 3, 6 and 22 hours after 2 mM IPTG induction. (A): Quantitative analysis of MamC-EGFP localization 0, 3 and 6 hours after induction. Fluorescent signals in around 200 cells where analyzed for each time point and classified into different localization patterns which are exemplified in representative micrographs of different time points in (B): Single bright spots (asterisk), bright spot and chain (asterisk + arrow), patches (hash) and chains (arrow). Scale bar: 5 μm. (TIF) [file pgen.1006101.s013.tif]

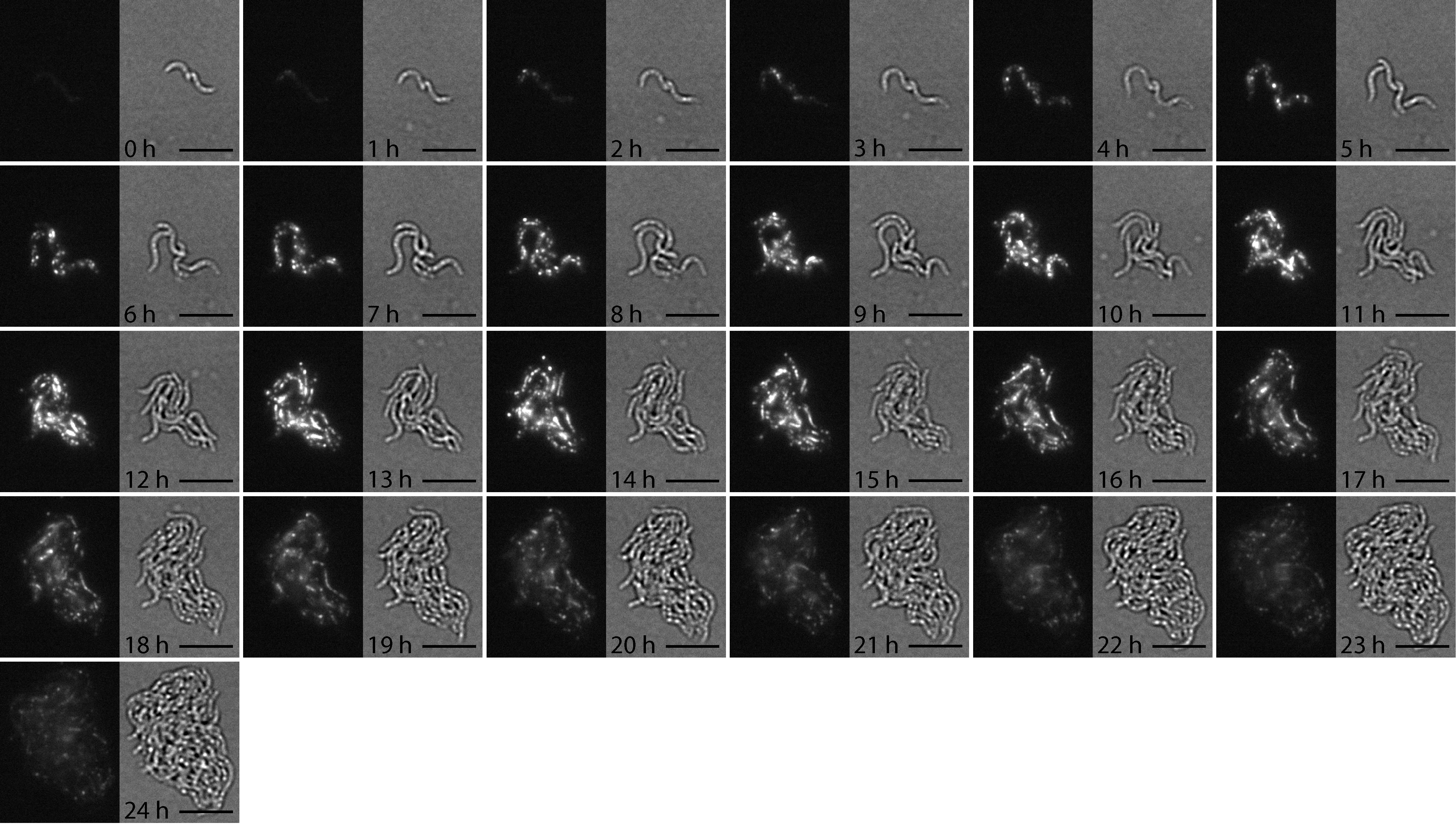

Supplement: S10 Fig — More detailed representation of Fig 6C. The strain was induced with 2 mM IPTG, transferred to 1% agarose pads containing modified FSM medium and 3 mM IPTG, sealed and incubated at 30°C. Images were acquired every 15 min. The depicted micrographs were acquired in 1 h intervals. Bright field and fluorescence channels are shown. Scale bar: 2 μm. (TIF) [file pgen.1006101.s014.tif]
