# Supplementary material for: Genetic and Ultrastructural Analysis Reveals the Key Players and Initial Steps of Bacterial Magnetosome Membrane Biogenesis
Source: PLoS Genet. 2016 Jun 10;12(6):e1006101. doi: 10.1371/journal.pgen.1006101 (PMC4902198; doi:10.1371/journal.pgen.1006101)
Supplement: S1 Table — MM: magnetosome membrane, DMM: dense magnetosome membrane-like structure. (DOCX) [file pgen.1006101.s025.docx]

**S1 Table:** Summary of phenotypes obtained by analyzing cryo-electron tomography data of different MSR-1 mutant cells. MM: magnetosome membrane, DMM: dense magnetosome membrane-like structure

| Strain | Total # of analyzed cells | # cells with chain of wild type-like MMs | # cells with dense chains of DMMs | # cells with single vesicular structures (DMMs or wild type-like) | # cells without vesicles |
| --- | --- | --- | --- | --- | --- |
| ∆*mamI* | 6 | 4 |  |  | 2 |
| ∆*mamN* | 5 | 4 |  |  | 1 |
| ∆*mamL* | 18 | 5* |  | 8 | 5 |
| ∆*mamQ* | 16 |  | 7** | 5*** | 4 |
| ∆*mamB* | 18 |  |  | 5**** | 13 |
| ∆*mamM* | 16 |  | 6** | 7*** | 3 |

* putative DMMs occasionally seen
** wildtype-like MMs occasionally seen
*** DMMs predominant
**** putative DMMs only
