# Supplementary material for: Genetic and Ultrastructural Analysis Reveals the Key Players and Initial Steps of Bacterial Magnetosome Membrane Biogenesis
Source: PLoS Genet. 2016 Jun 10;12(6):e1006101. doi: 10.1371/journal.pgen.1006101 (PMC4902198; doi:10.1371/journal.pgen.1006101)
Supplement: S2 Table — (DOCX) [file pgen.1006101.s026.docx]

S2 Table: Bacterial strains used in this study

| Strains and plasmids | Description | Source |
| --- | --- | --- |
| MSR strains |  |  |
| MSR-1 R3/S1 | wildtype and mother strain of deletion mutants | [15] |
| Δ*mamAB* | Cre/lox deletion | [16] |
| 1B | Spontaneous partial MAI deletion mutant, including deletion of *mamAB*, *mms6*, and *mamGFDC* operons (non-magnetic) | [17] |
| Δ*mamI* | Markerless in-frame deletion | [18] |
| Δ*mamN* | Markerless in-frame deletion | [18] |
| Δ*mamQ* | Markerless in-frame deletion | [18] |
| Δ*mamL* | Markerless in-frame deletion | [18] |
| Δ*mamB* | Cre/lox deletion | [12] |
| Δ*mamM* | Cre/lox deletion | [12] |
| Δ*mamL* P*_lac_*-*mamL, lacI* (MamL_ind_) | Tn7 transposon integrated (pOR118) | this study |
| Δ*mamB* P*_lac_*_-_*mamB, lacI* (MamB_ind_) | Tn7 transposon integrated (pOR158) | this study |
| Δ*mamB* P*_lac_*_-_*mamB-egfp, lacI* (MamB-EGFP_ind_) | Tn7 transposon integrated (pOR169) | this study |
| *mamQ::mCherry*:*mamQ* | Allelic replacement (pYF002) | this study |
| *mamQ::egfp*-*mamQ* | Allelic replacement (pYF001) | this study |
| *mamQ::egfp*-*mamQ*_[Y242A F242A]_ | Allelic replacement (pYF003) | this study |
| *mamQ::egfp*-*mamQ*_[E179A]_ | Allelic replacement (pYF004) | this study |
| *mamQ::egfp*-*mamQ*_[Y181A]_ | Allelic replacement (pYF005) | this study |
| *mamQ::egfp*-*mamQ*_[E111A]_ | Allelic replacement (pYF006) | this study |
| *mamQ::egfp-mamQ*_[E179A Y181A E111A]_ | Allelic replacement (pYF007) | this study |
| Δ*mamQ* P*_mamDC_-egfp-mamQ* | Tn5 transposon integrated | this study |
| *mamQ::mCherry*-*mamQ mamB::mamB:egfp* | Allelic replacements (pORFM B-GFP, pYF002) | this study |
| Δ*mamL* P*_mamDC_-mamL-egfp* | Tn5 transposon integrated (pOR151**)** | this study |
| Δ*mamL* P*_mamDC_-mamL*_[K77Q R78Q]_*-egfp* | Tn5 transposon integrated (pOR163**)** | this study |
| Δ*mamL* P*_mamDC_-mamL*_[K72Q]_*-egfp* | Tn5 transposon integrated (pOR164**)** | this study |
| Δ*mamL* P*_mamDC_-mamL*_[K63Q]_*-egfp* | Tn5 transposon integrated (pOR165**)** | this study |
| Δ*mamL* P*_mamDC_*-*mamL*_[K63Q K66Q K68Q]_*-egfp* | Tn5 transposon integrated (pOR166**)** | this study |
| Δ*mamL* P*_mamDC_*-*mamL*_[R64Q R65Q]_ *-egfp* | Tn5 transposon integrated (pOR167**)** | this study |
| Δ*mamL* P*_mamDC_*-*mamL*_[all neutral]_ *-egfp* | Tn5 transposon integrated (pOR168**)** | this study |
| Δ*mamAB* P*_mamAB_-mamLQRB* | Tn5 transposon integrated (pOR140) | this study |
| 1B P*_mamAB_-mamLQRB* | Tn5 transposon integrated (pOR140) | this study |
| 1B P*_mamAB_-mamLMQRB* | Tn5 transposon integrated (pOR155) | this study |
| Δ*mamL* P*_mamAB_-mamLMQRB* | Tn5 transposon integrated (pOR155) | this study |
| Δ*mamQ* P*_mamAB_-mamLMQRB* | Tn5 transposon integrated (pOR155) | this study |
| Δ*mamB* P*_mamAB_-mamLMQRB* | Tn5 transposon integrated (pOR155) | this study |
| Δ*mamM* P*_mamAB_-mamLMQRB* | Tn5 transposon integrated (pOR155) | this study |
| Δ*mamAB* P*_mamAB_-mamLQB-* P*_mamAB_-mamIEMO* | Tn5 transposon integrated (pBAM_minMAI) | this study |
| 1B P*_mamAB_-mamLQB-* P*_mamAB_-mamIEMO* | Tn5 transposon integrated (pBAM_minMAI) | this study |
| Δ*mamQ* P*_mamAB_-mamLQB-* P*_mamAB_-mamIEMO* | Tn5 transposon integrated (pBAM_minMAI) | this study |
| Δ*mamM* P*_mamAB_-mamLQB-* P*_mamAB_-mamIEMO* | Tn5 transposon integrated (pBAM_minMAI) | this study |
| *E. coli* strains |  | this study |
| BW29427 | *thrB1004 pro thi rpsL hsdS lacZDM15 RP4-1360D(araBAD)567DdapA* | Datsenko & Wanner, unpublished |
| DH5a | *1341::[erm pir(WT)]trahsdR17 recA1-endA1gyrA96thi-1relA1* | Invitrogen |
| S17-1*λpir* | RPA-2, Tc::Mu-Km::Tn7 (λpir) | [19] |
| *WM3064* | *thrB1004 pro thi rpsL hsdS lacZDM15 RP4-1360D(araBAD) 567DdapA*::[*erm pir*] | W. Metcalf, unpublished |
