# Supplementary material for: Genetic and Ultrastructural Analysis Reveals the Key Players and Initial Steps of Bacterial Magnetosome Membrane Biogenesis
Source: PLoS Genet. 2016 Jun 10;12(6):e1006101. doi: 10.1371/journal.pgen.1006101 (PMC4902198; doi:10.1371/journal.pgen.1006101)
Supplement: S3 Table — (DOCX) [file pgen.1006101.s027.docx]

S3 Table: Plasmids used in this study

| Plasmids |  |  |
| --- | --- | --- |
| pJet1.2 | Ap^r^, *eco47IR*, *rep* (pMB-1) | Life Technologies |
| pBAM1 | *ori*R6K, Km^r^, Ap^r^ | [23] |
| pBBR1MCS2 | Mobilizable broad-host-range vector, Km^R^ | [24] |
| pT18mob2 | Mobilizable broad-host-range vector, Km^R^ | [25] |
| pUC18R6K-mini-Tn7T-Km | Mini-Tn7 transposable plasmid, Km^r^ | [26] |
| pTns2 | Tn7 transposase helper plasmid | [26] |
| pT18-Tn7T-Km | MSR adapted mini-Tn7 transposable plasmid, Km^r^ | this study |
| pT18mob2PmamDC-TnsAD | MSR adapted Tn7 transposase helper plasmid | this study |
| pORFM GalK | Vector for genomic in-frame deletions/integration | [5] |
| pORFM blu | Vector for genomic in-frame deletions/integration | [5] |
| pFM211 | pBBR1 P*_lac_*-*ftsZm-mCherry*, *egfp-mamK*, *lacI*, Km^r^ | [10] |
| pOR071 | pBBR1 P*_mamDC_*-*egfp*, Km^r^ | [27] |
| pOR075 | pBBR1 P*_mamDC_*-*mamI-egfp*, Km^r^ | this study |
| pOR077 | pBBR1 P*_mamDC_*-*mamL-egfp*, Km^r^ | this study |
| pOR082 | pBBR1 P*_mamDC_*- RR(MGR0500)-*egfp*, Km^r^ | this study |
| pOR086 | pBBR1 P*_mamDC_*-*egfp-mamQ*, Km^r^ | this study |
| pOR117 | pBBR1 P*_lac_*-*mamL*, *lacI*, Km^r^ | this study |
| pOR118 | pT18-Tn7T-Km P*_lac_*-*mamL*, *lacI*, Km^r^ | this study |
| pOR140 | pBAM P*_mamAB_*-*mamLQRB*, Km^r^ | this study |
| pOR150 | pBAM P*_mamDC_*-*egfp-mamQ*, Km^r^ | this study |
| pOR151 | pBAM P*_mamDC_*-*mamL-egfp*, Km^r^ | this study |
| pOR155 | pBAM P*_mamAB_*-*mamLMQRB*, Km^r^ | this study |
| pOR158 | pBBR1 P*_lac_*-*mamB*, *lacI*, Km^r^ | this study |
| pOR160 | pT18-Tn7T-Km P*_lac_*-*mamB*, *lacI*, Km^r^ | this study |
| pOR163 | pBAM P*_mamDC_*-*mamL*_[K77Q R78Q]_*-egfp*, Km^r^ | this study |
| pOR164 | pBAM P*_mamDC_*-*mamL*_[K72Q]_*-egfp*, Km^r^ | this study |
| pOR165 | pBAM P*_mamDC_*-*mamL*_[K63Q K66Q K68Q]_*-egfp*, Km^r^ | this study |
| pOR166 | pBAM P*_mamDC_*-*mamL*_[H67Y ]_*-egfp*, Km^r^ | this study |
| pOR167 | pBAM P*_mamDC_*-*mamL*_[R64Q R65Q]_*-egfp*, Km^r^ | this study |
| pOR168 | pBAM P*_mamDC_*-*mamL*_[K63Q R64Q R65Q K66Q H67Y K68Q K72Q K77Q R78Q (all neutral)]_*-egfp*, Km^r^ | this study |
| pOR169 | pBBR1 P*_lac_*-*mamB-GFP*, *lacI*, Km^r^ | this study |
| pOR171 | pT18-Tn7T-Km P*_lac_*-*mamB-egfp*, *lacI*, Km^r^ | this study |
| pYF001 | pORFM derivate, for chromosomal *eGFP*-*mamQ* in-frame fusion, Km^r^ | this study |
| pYF002 | pORFM derivate, for chromosomal *mCherry*-*mamQ* in-frame fusion, Km^r^ | this study |
| pYF003 | pORFM derivate, for chromosomal *eGFP*-*mamQ*_[Y242A F242A]_ in-frame fusion, Km^r^ | this study |
| pYF004 | pORFM derivate, for chromosomal *eGFP*-*mamQ*_[E179A]_ in-frame fusion, Km^r^ | this study |
| pYF005 | pORFM derivate, for chromosomal *eGFP*-*mamQ*_[Y181A]_ in-frame fusion, Km^r^ | this study |
| pYF006 | pORFM derivate, for chromosomal *eGFP*-*mamQ*_[E111A]_ in-frame fusion, Km^r^ | this study |
| pYF007 | pORFM derivate, for chromosomal *eGFP*-*mamQ*_[E179A Y181A E111A]_ in-frame fusion, Km^r^ | this study |
| pORFM B-GFP | pORFM derivate, for chromosomal *mamB-GFP* in frame-fusion, Km^r^ | this study |
| pA0-mamX-Tn5 | Plasmid harboring fully synthetic P*_mamAB_*-*mamLBQ* P*_mamAB_*-*mamIEMO* expression cassette, Km^r^ | this study |
| pBAM_minMAI | pBAM P*_mamAB_*-*mamLBQ* P*_mamAB_*-*mamIEMO*, Km^r^ | this study |
