# Supplementary material for: Genetic and Ultrastructural Analysis Reveals the Key Players and Initial Steps of Bacterial Magnetosome Membrane Biogenesis
Source: PLoS Genet. 2016 Jun 10;12(6):e1006101. doi: 10.1371/journal.pgen.1006101 (PMC4902198; doi:10.1371/journal.pgen.1006101)
Supplement: S4 Table — (DOCX) [file pgen.1006101.s028.docx]

S4 Table: Oligonucleotides used in this study

| Identifier | Description/Name | Sequence |
| --- | --- | --- |
| oOR113 | oegfp_rw | CCATCTCGAGTCACTTATACAGCTCGTCCATGCC |
| oOR134 | oegfp_fw | ATTAGGATCCGTGTCGAAGGGCGAGGAACTG |
| oOR130 | MGR0500_TAT_fw | ACTGCATATGTACGACGACAGCGCCG |
| oOR146 | MGR0500_TAT_rev | TCTAGGATCCGATGGCGGCGGCGATCTG |
| oOR164 | Oligo for Helix linker_fw | TACTCGAGGCTAGCCTGGCCGAAGCCGCGGCCAAGGAGGCCGCCGCGAAGGAAGCCGCGGCCAAGGAGGCCGCCGCGAAGGCCGCGGCCGTGCACGGTACCTA |
| oOR165 | Oligo for Helix linker_rev | TAGGTACCGTGCACGGCCGCGGCCTTCGCGGCGGCCTCCTTGGCCGCGGCTTCCTTCGCGGCGGCCTCCTTGGCCGCGGCTTCGGCCAGGCTAGCCTCGAGTA |
| oOR166 | oegfp_fw (ApaLI, MfeI) | TACTGTGCACATGGTGTCGAAGGGCGAGG |
| oOR167 | oegfp_rw (ApaLI, MfeI) | TAGTCAATTGTCACTTATACAGCTCGTCCATGCC |
| oOR112 | o_egfp_fw | TACGCATATGGTGTCGAAGGGCGAGG |
| oOR169 | oegfp_wo stop_rev | ATCACTCGAGCTTATACAGCTCGTCCATGCCC |
| oOR170 | mamI_fw | GATACATATGCCAAGCGTGATTTTCGG |
| oOR155 | mamI_rev | ATACGCTAGCACCATCGATGTTAGGGTCTGAGTTC |
| oOR171 | mamL_fw | AGCTCATATGGTAAGAGTGATCGGATCGTTGG |
| oOR157 | mamL rev | TGTGGCTAGCGCGCTTAATGACGATGTTTTTCCC |
| oOR158 | mamL-NdeI fw | CCCGATTTGGGCCACTGATAATGC |
| oOR159 | mamL-NdeI rev | TTTCCACCATGTGGGCGTTGG |
| oOR181 | mamQ_fw | ACTCCATATGGTGCACGCAGTAAGCGATGCGGACG |
| oOR182 | mamQ_rev | ACTCATGCATTCAGCTAGCATTCTTGGATTCCTGCGAATGG |
| oOR199 | mamQ-NdeI fw | CCTACAAGCACATGATGACCTCG |
| oOR200 | mamQ-NdeI rev | TATCCGCCGACTGAACGG |
| oOR264 | mamQ-down_fw | TAGAGTCGACCCGATTGCCATCCGGTAGG |
| oOR265 | mamQ-down_rev | CCTCGCCCTTCGACACCATATCCGCCTCGTTGCTATCGTC |
| oOR266 | GFP_fw | GACGATAGCAACGAGGCGGATATGGTGTCGAAGGGCGAGG |
| oOR267 | mamQ_rev_stopp | ACTCACTAGTTCAATTCTTGGATTCCTGCGAATGG |
| oOR278 | mcherry_mamQ_fw (overlap extension) | TGAGATGACGATAGCAACGAGGCGGATATGGTGAGCAAGGGCGAGG |
| oOR279 | mcherry_mamQ_rev | CGGCTTCGGCCAGGCTAGCCTCGAGCTTGTACAGCTCGTCCATGCC |
| oOR282 | mamL_rev | CTAGCTCGAGTCAGCGCTTAATGACGATGTTTTTCC |
| oOR283 | Plac_Tn7_fw | GACTAAGCTTCGTTGGCCGATTCATTAATGC |
| oOR284 | Plac_Tn7_rev | TAGAGAGCTCGGAACAAAAGCTGGGTACCC |
| oOR290 | mamQ_up_fw | CTGAGTCGACCCACGTGATAAATTAGCCAGAGCC |
| oOR291 | mamQ_up_rev | CATAGCTAGCCCGCCTTTCCGATCAATTCC |
| oOR296 | Q_TyrPhe_AlaAla forw | TGGAGGCTGCCCACGAAAAGC |
| oOR297 | Q_TyrPhe_AlaAla rev | CGCGCTTGAACCCGGTG |
| oOR298 | Q_Glu112_Ala fw | CCATTGTCGCGCAGTATCCC |
| oOR299 | Q_Glu112_Ala rev | CCATCAGCCGCCCCAGC |
| oOR300 | Q_Tyr114_Ala fw | CGAGCAGGCTCCCACCG |
| oOR301 | Q_Tyr114_Ala rev | ACAATGGCCATCAGCCGC |
| oOR302 | Q_Glu44_Ala fw | CCGCTTTGGCGCACTCCATC |
| oOR303 | Q_Glu44_Ala rev | CATGATTCAGCGTCAACTTGACC |
| oOR304 | Q_GluTyr_AlaAla fw | CATTGTCGCGCAGGCTCCCACC |
| oOR305 | Q_GluTyr_AlaAla rev | GCCATCAGCCGCCCCAGC |
| oOR357 | PmamAB_fw | CTAGAttatggcttgtcaaccgacctcgattcgttgctatagtccgtgcgaattggaggtgaattgtgacgCA |
| oOR358 | PmamAB_rev | TATGcgtcacaattcacctccaattcgcacggactatagcaacgaatcgaggtcggttgacaagccataaT |
| oOR359 | mamL_rev | AGTAGCTAGCTCAGCGCTTAATGACGATGTTTTTCC |
| oOR360 | mamQ_5UTR_fw | AGTAGCTAGCGATGTAGAAGGTCTCATTGAGATGACG |
| oOR361 | mamB_rev | ACTCATGCATTCAGACCCGGACCGTCACG |
| oOR375 | MamM_rev | AGTAGCTAGCCTAGTTATCCACCTTGGACAGCATGAC |
| oOR386 | mamB_fw | ACTGCATATGAAGTTCGAAAATTGCAGAGACTGC |
| oOR387 | mamB_rev | ATCACTCGAGTCAGACCCGGACCGTCACG |
| oOR396 | mamL_K5R5-QQ_GFP_fw | CATCGTCATTCAGCAAGCTAGCCTGGC |
| oOR397b | mamL_K5R5-QQ_rev | TTTTTCCCAGGCAGTTTATGCTTGC |
| oOR398 | mamL_K4-Q_fw | CTGCCTGGGCAGAACATCGTC |
| oOR399 | mamL_K4-Q_rev | TTTATGCTTGCGCCGTTTCATAAC |
| oOR400 | mamL_K1K2K3-QQQ_fw | GCGCCAGCATCAACTGCCTG |
| oOR401 | mamL_K1K2K3-QQQ_rev | CGTTGCATAACATTCGCAAACAC |
| oOR402 | mamL_H3-Y_fw | GGCGCAAGTATAAACTGCCTGG |
| oOR403 | mamL_H3-Y_rev | GTTTCATAACATTCGCAAACACCAAC |
| oOR404 | mamL_R3R4-QQ_fw | ATGTTATGAAACAGCAGAAGCATAAACTGC |
| oOR405 | mamL_R3R4-QQ_rev | TCGCAAACACCAACACAATCC |
| oOR420 | mamB_fw | ACTGCATATGAAGTTCGAAAATTGCAGAGACTG |
| oOR421 | mamB_rev | GCTAGCGACCCGGACCGTCACGG |
| oOR422 | Helix-oegfp_fw | GCTAGCCTGGCCGAAGCC |
| oOR423 | Helix-oegfp_rev | TGCGGTCGACTCACTTATACAGCTCGTCCATGCC |
| oOR435 | MluI_ins_fw | CTAGACCTAACGCGTAATCGAGCT |
| oOR436 | MluI_ins_rev | CGATTACGCGTTAGGT |
|  | pT18mob2+Tet_for_NotI | CATGGCGGCCGCAGTCATAGTTGCACTTTATCA |
|  | pT18mob2_rev_NcoI | CATCCCATGGCCAGTCGGGAAACCTGTCGT |
|  | Tn7Km_for | CAGCCGCGTAACCTGGCAAA |
|  | Tn7Km_rev | CAGTGAGCGAGGAAGCGGAA |
|  | TnsA for | ACACTTGCCGGAGATCAGATGGCTAAAGCAAACTCTT |
|  | TnsA rev | TTAATTTGCCACATAGCG |
|  | PmamDCrev | AAGAGTTTGCTTTAGCATGCTGATCTCCGGCAAGTGTATG |
|  | PmamDC96 | CTTTTTCGCTTTACTAGCTC |
|  | mamB(wt)_KpnI_for | CGGCGGTACCATGAAGTTCGAAAATTGC |
|  | HL3-oeGFP-mamBC-term_for | TGACGGTCCGGGTCTGGTGCTAGCCTGGCCGAAGCC |
|  | HL3-oeGFP-mamBC-term_rev | GAGCTCGGCTTCCATGTCGGCAGA |
|  | mamBC-termoeGFP_rev | GCGGCTTCGGCCAGGACCCGGACCGTCACGGC |
|  | oeGFP_Bdo_for | agctgtataagtgaTCAGGAGAGGGGAATCATGGAC |
|  | oeGFP_Bdo_rev | TTCCCCTCTCCTGATCACTTATACAGCTCGTCC |
